# Exposure of Platelets to Dengue Virus and Envelope Protein Domain III Induces Nlrp3 Inflammasome-Dependent Platelet Cell Death and Thrombocytopenia in Mice

**DOI:** 10.3389/fimmu.2021.616394

**Published:** 2021-04-29

**Authors:** Te-Sheng Lien, Hao Chan, Der-Shan Sun, Jhen-Cheng Wu, You-Yen Lin, Guan-Ling Lin, Hsin-Hou Chang

**Affiliations:** Department of Molecular Biology and Human Genetics, Tzu-Chi University, Hualien, Taiwan

**Keywords:** dengue hemorrhage fever, platelet, Nlrp3 inflammasome, envelope protein domain III, pyroptosis, necroptosis, ferroptosis, apoptosis

## Abstract

In tropical and subtropical regions, mosquito-borne dengue virus (DENV) infections can lead to severe dengue, also known as dengue hemorrhage fever, which causes bleeding, thrombocytopenia, and blood plasma leakage and increases mortality. Although DENV-induced platelet cell death was linked to disease severity, the role of responsible viral factors and the elicitation mechanism of abnormal platelet activation and cell death remain unclear. DENV and virion-surface envelope protein domain III (EIII), a cellular binding moiety of the virus particle, highly increase during the viremia stage. Our previous report suggested that exposure to such viremia EIII levels can lead to cell death of endothelial cells, neutrophils, and megakaryocytes. Here we found that both DENV and EIII could induce abnormal platelet activation and predominantly necrotic cell death pyroptosis. Blockages of EIII-induced platelet signaling using the competitive inhibitor chondroitin sulfate B or selective Nlrp3 inflammasome inhibitors OLT1177 and Z-WHED-FMK markedly ameliorated DENV- and EIII-induced thrombocytopenia, platelet activation, and cell death. These results suggest that EIII could be considered as a virulence factor of DENV, and that Nlrp3 inflammasome is a feasible target for developing therapeutic approaches against dengue-induced platelet defects.

## Introduction

Dengue, caused by dengue virus (DENV), is a commonly observed mosquito-borne infectious disease in the tropical and subtropical areas of the world (1, 2). Currently, no specific antiviral treatment and reliable vaccine are available against dengue (3–8). Over half of the global population lives in areas with DENV infection risk, resulting in 96 million patients with symptomatic dengue every year (9). The primary infection of DENV is a self-limiting dengue fever (DF), whereas secondary infections are epidemiologically associated with an increased risk of life-threatening severe dengue [also known as dengue hemorrhage fever (DHF)]. Thrombocytopenia and platelet defects are the frequently observed symptoms in both primary and secondary dengue infection; however, the responsible pathogenic factor remains unclear. Here we hypothesize that the DENV envelope protein (E) domain III (EIII) can be one of the viral factors responsible for pathological changes in platelets.

The DENV EIII is an Ig-like domain involved in host cell receptor binding for viral entry (10). However, whether cellular signaling occurs after EIII binds to target cells remains unclear. Pieces of evidence have shown that DENV-induces activation of inflammasome complexes (11, 12). Our previous study revealed that challenges with the DHF-viral-load-equivalent levels of EIII can induce cell death in endothelial cells (13) and neutrophils (14), and suppress megakaryopoiesis through autophagy impairment and apoptosis (15). These results suggest that EIII binding to platelets could be cytotoxic, particularly when circulating virion-surface EIII reaches the peak of the viremia stage. In addition, because gene deficiencies in Nlrp3 inflammasome components display protective effects on the hemorrhage fever mouse model (13, 16), we further investigated whether Nlrp3 inflammasome contributes to EIII-induced platelet defects. In this study, EIII treatments directly caused the activation and death of platelets *in vitro* and thrombocytopenia in mice. Intriguingly, pyroptosis is the major regulated cell death (RCD) pathway of DENV- and EIII-treated platelets. In addition, treatment with the Nlrp3 inflammasome selective inhibitors OLT1177 and Z-WHED-FMK markedly ameliorated DENV- and EIII-induced platelet defects *in vitro* and *in vivo*, suggesting that the Nlrp3 inflammasome can be a feasible target for treating dengue-associated thrombocytopenia and platelet defects. Related implications and applications are discussed.

## Materials and Methods

### DENV, Recombinant Protein, and Antibodies

The soluble recombinant proteins DENV EIII (rEIII), non-structural protein 1 (rNS1) and glutathione-S transferase (rGST), and DENV-2 virus particle (strain PL046) were obtained using previously described methods (15–19). DENV-EIII DNA (plasmid DENV-2EIII/pET21b; amino acids 578–674) was kindly provided by Prof. Yi-Ling Lin (Institute of Biomedical Sciences, Academia Sinica, Taiwan). The rEIII protein was obtained using nickel-nitrilotriacetic acid metal-affinity chromatography (Qiagen, Qiagen Taiwan, Taipei, Taiwan). To the column washing buffer (8 M urea, 100 mM NaH_2_PO_4_, and 10 mM Tris–HCl; pH = 6.3), 1% Triton X-114 (Sigma–Aldrich (20); was added to remove the endotoxin (lipopolysaccharide; LPS) to a desired level (<1 EU/mg protein). As previously described (15), rNS1 and rEIII were eluted with a buffer (8 M urea, 100 mM NaH_2_PO_4_, and 10 mM Tris–HCl; pH = 4.5) and refolded using a linear 4–0 M urea gradient in a dialysis buffer (2 mM reduced glutathione, 0.2 mM oxidized glutathione, 80 mM glycine, 1 mM ethylenediaminetetraacetic acid, 50 mM Tris–HCl, 50 mM NaCl, and 0.1 mM phenylmethylsulfonyl fluoride) at 4°C for 2–3 h. The rEIII protein was purified to approximately 90% (**Figure S1**). Using flow cytometry, the proper folding of EIII, which enables its platelet-binding properties, was double checked through comparisons to heat-inactivated control proteins (**Figure S2**). The Limulus Amoebocyte Lysate QCL-1000 kit (Lonza, Walkersville, MD, USA) (19, 21, 22) was used to measure and monitor the remaining LPS levels of each purification batch of recombinant proteins, and batches with an LPS contamination level of <1 EU/mg of protein were used. Other recombinant DENV-2 envelope protein fragments used in platelet-binding experiments (45 kDa domains I + II + III, 32 kDa domains I + II, and 25 kDa domains II c-terminal + III) were purchased from ProSpec-Tany TechnoGene (Ness-Ziona, Israel). The anti-EIII sera were obtained after the third immunization cycle. The IgG fractions were obtained and purified using a protein-A column attached to a peristaltic pump (Amersham Biosciences, flow rate of 0.5–1 ml min^−1^) and were subsequently washed and eluted (16). For competition between rEIII- and rNS1-platelet binding, the following recombinant proteins were used, recombinant mouse C-type lectin domain family 5 member A (CLEC5A), mouse CD42b/GPIb *α*, human TLR4 (R&D Systems).

### Experimental Mice

Wild-type C57BL/6J mice aged 8–12 weeks were purchased from the National Laboratory Animal Center (Taipei, Taiwan) (17, 23–27). Genetically deficient mice with a C57BL/6J background, including *Nlrp3* ^−^ *^/^* ^−^ and *Casp1* ^−^ *^/^* ^−^, were obtained from the Centre National de Recherche Scientifique (Orléans, France) (16, 28). All animals were housed in the Animal Center of Tzu-Chi University in a specific pathogen-free temperature- and light-controlled environment with free access to filtered water and food. All genetic knockout strains were backcrossed with wild-type C57Bl/6J mice for at least six generations.

### Ethics Statement

Animal experiments in this study were conducted in agreement with the National (Taiwan Animal Protection Act, 2008) directive for the protection of laboratory animals. All experimental protocols for examining experimental animals were approved by the Animal Care and Use Committee of Tzu-Chi University, Hualien, Taiwan (approval ID: 101019).

### Blood and Platelet Isolation and Parameter Analyses

Mouse blood samples were collected through the retro-orbital venous plexus technique by using plain capillary tubes (Thermo Fisher Scientific, Waltham, MA, USA) and were transferred into polypropylene tubes containing anticoagulant acid-citrate-dextrose solution (38 mM citric acid, 75 mM sodium citrate, and 100 mM dextrose) (29, 30). Washed platelets were prepared as previously described (31, 32). Platelet-poor plasma was prepared through centrifugation at 1,500 × g for 20 min. Platelet-poor plasma was centrifuged again for 3 min at 15,000 × g to remove contaminant cells from the plasma. According to manufacturer’s instructions, the activated partial thromboplastin time (aPTT) experiment was performed using a coagulometer (ACL-Futura Plus, Instrumentation Laboratory, Milan, Italy; (29, 33). The red blood cell and platelet counts of mice were measured using a hematology analyzer (KX-21N; Sysmex, Kobe, Japan) (26).

### Analyses of Platelet Response on Viral-Protein Exposure

To analyze the binding properties of rEIII proteins on platelets, rEIII proteins (laboratory-prepared 20-kDa domain III, 45-kDa domains I + II + III, 32-kDa domains I + II, and 25-kDa domains II c-terminal + III purchased from ProSpec-Tany TechnoGene) were conjugated with biotin by using an EZ-Link™ Sulfo-NHS-Biotinylation kit (Thermo Fisher Scientific). The levels of biotin-labeled rEIII proteins bound to mouse platelets (50 μg/ml protein + 1 × 10^7^ platelets/ml in Tyrodes buffer for 30 min) were determined through flow cytometry by using PE/Cy5 avidin (Biolegend, San Diego, CA, USA) staining. The rEIII-competitive inhibitor chondroitin sulfate B (CSB, 10 µg/ml; Sigma-Aldrich, St. Louis, MO, USA) was used to suppress rEIII-induced platelet activation. The fluorescence intensities of rEIII-bound platelets were analyzed using a fluorescent microplate reader (Varioskan™ Flash Multimode reader; Thermo Fisher Scientific) (26, 29) at Excitation/Emission spectrum = 480/530 nm. A flow cytometer (FACScalibur, BD Biosciences) (29, 30) was used for analyzing platelet surface P-selectin expression and biotin-rEIII binding by using anti-mouse P-selectin Ig-phycoerythrin (PE; eBioscience, Thermo Fisher Scientific) and biotin-conjugated rEIII, followed by avidin-PE/Cy5 (Biolegend) labeling. Scanning electron microscopy analysis for platelet morphology was conducted as previously described (31). The washed mouse platelets were incubated for 15 min under coverslips coated with various amounts of rEIII or rGST. After washing with phosphate-buffered saline (PBS), these cells were fixed with glutaraldehyde and subjected to a series of alcohol dehydrations, critical point drying procedures, and gold coating. The platelet morphology of the cells was then observed under a scanning electron microscope at 15 kV (Hitachi S-4700, Hitachi, Japan). Morphological scoring and quantification of platelet activation levels were conducted using previously described methods (34). Six different areas were randomly selected for photography at each magnification for quantitative analyses. Mouse bleeding time was measured 24 h after rEIII injections with or without CSB rescues (0.5 mg/kg).

### Platelet Cell Death and Mitochondrial Analyses

To analyze DENV- or rEIII-induced platelet cell death, washed mouse platelets were incubated with DENV or rEIII for 1 h and then subjected to flow cytometry analyses after washing with PBS. Various RCD responses, namely including apoptosis (CaspGLOWTM Red Active Caspase-3 Staining Kit, BioVision, Milpitas, CA, USA), autophagy (Cyto-ID™ Autophagy Detection Kit, Enzo Life Sciences, Farmingdale, NY, USA), ferroptosis (C11 BODIPY 581/591, Cayman Chemical, Ann Arbor, MI, USA), necroptosis (RIP3/B-2 alexa Fluor 488, Santa Cruz Biotechnology, Santa Cruz, CA, USA), pyroptosis [Caspase-1 Assay, Green-Fluorochrome-Labeled Inhibitors of Caspases (FLICA), ImmunoChemistry Technologies, MI, USA; Caspase-1 Colorimetric Assay Kit, BioVision, Milpitas, CA, USA; flow cytometry, antibody against gasdermin D (GSDMD), Abcam, Cambridge, UK], and live/dead cell labeling (Zombie NIR™ Fixable Viability Kit, Biolegend), cell death detection [Lactate Dehydrogenase (LDH) Assay Colorimetric Kit, Abcam], were analyzed using respective cell labeling reagents (30 min in PBS). Treatments (1 h) of cell death inducers were used as positive controls for various types of RCD [apoptosis: doxorubicin, 2.5 μg/ml, Nang Kuang Pharmaceutical, Taipei, Taiwan; autophagy: rapamycin, 250 nM, Sigma–Aldrich; ferroptosis: erastin, 10 μM, Cayman Chemical; necroptosis: tumor necrosis factor alpha (TNF-*α*), 2 ng/ml, Biolegend; pyroptosis: nigericin, 3.5 μM, ImmunoChemistry Technologies, Minnesota, USA; 30 min in PBS]. Inhibitors were used to address the involvement of specific RCD pathways [apoptosis: Z-DEVD-FMK, 10 μM, R&D Systems, Indianapolis, IN, USA; autophagy: chloroquine diphosphate, 60 μM, Sigma–Aldrich; ferroptosis: ferrostatin-1, 2.5 μM, Cayman Chemical; necroptosis: necrostatin-1, 50 μM, Cayman Chemical; pyroptosis: Z-WHED-FMK, 10 μM, R&D Systems, dimethyl fumarate (DMF), 50 μM, Sigma–Aldrich; inflammasome: OLT1177, 10 μM, Cayman Chemical; 1 h pretreatments before addition of rEIII, and cell-death inducers]. To analyze the induction of mitochondrial superoxide, MitoSOX™ Red mitochondrial superoxide indicator was used (Thermo Fisher Scientific; 30 min in PBS). Levels of interleukin (IL)-1*β* were determined through enzyme-linked immunosorbent assay (ELISA) (Biolegend) 30 min after rEIII treatments.

### Western Blotting

Western blotting analysis was performed following previously described methods (28, 35–38). A mini trans-blot electrophoretic transfer cell, a vertical electrophoresis gel tank, and a power pac 200 electrophoresis power supply (Bio-Rad Laboratories, Hercules, CA, USA) were used to separate and transfer the platelet protein samples to a polyvinylidene fluoride membrane (Immobilon-P, 0.45 µm, Merck Millipore, Burlington, MA, USA). Pre-stained protein ladders (PageRuler, Thermo Fisher Scientific) and an antibody against GSDMD (Abcam) were used to mark the molecular weight position and to stain the GSDMD, respectively. The intensities of Western-blotting image were measured using ImageJ software (version 1.32; National Institutes of Health, USA).

### Statistical Analyses

Using SigmaPlot 10 (Systat Software, San Jose, CA, USA) and SPSS 17 software packages (International Business Machines Corporation, Armonk, NY, USA), the means, standard deviations, and statistics of quantifiable data presented in this report were calculated. Using one-way analysis of variance and the *post-hoc* Bonferroni-corrected *t* test, the significance of data was examined. The probability of type-1 error *α* = 0.05 was set as the threshold of statistical significance.

## Results

### Soluble Recombinant EIII Protein Binds to Platelets and Induces Coagulation Defects, Platelet Activation, and Thrombocytopenia in Mice

**Figure 1:**
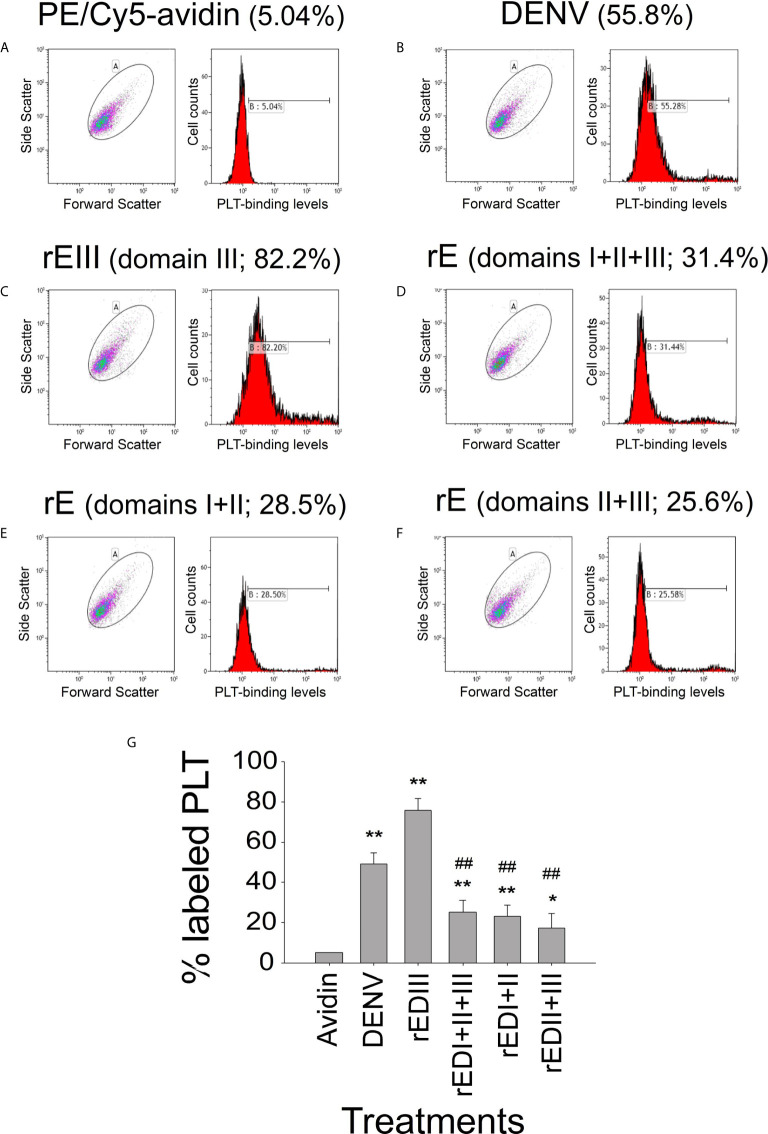
Platelet-binding property of DENV and different recombinant DENV envelope protein (rE) fragments. Flow cytometry analysis of the platelet-binding properties of biotinylated DENV and recombinant DENV envelope proteins (rEs) through PE/Cy5-avidin labeling. **(A)** Platelet PE/Cy5-avidin binding signal served as the background level. The respective blots and histograms on the platelet-binding of **(B)** DENV, **(C)** rE (domain III), **(D)** rE (domain I + II + III), **(E)** rE (domain I + II), and **(F)** rE (domain II + III) were indicated, and **(G)** quantified. n = 6, *P < 0.05, **P < 0.01 *vs.* avidin groups; ^##^ *P* < 0.01 *vs.* rEIII groups. DENV2 PL046 viral particles were used in this and the following figures.

**Figure 2:**
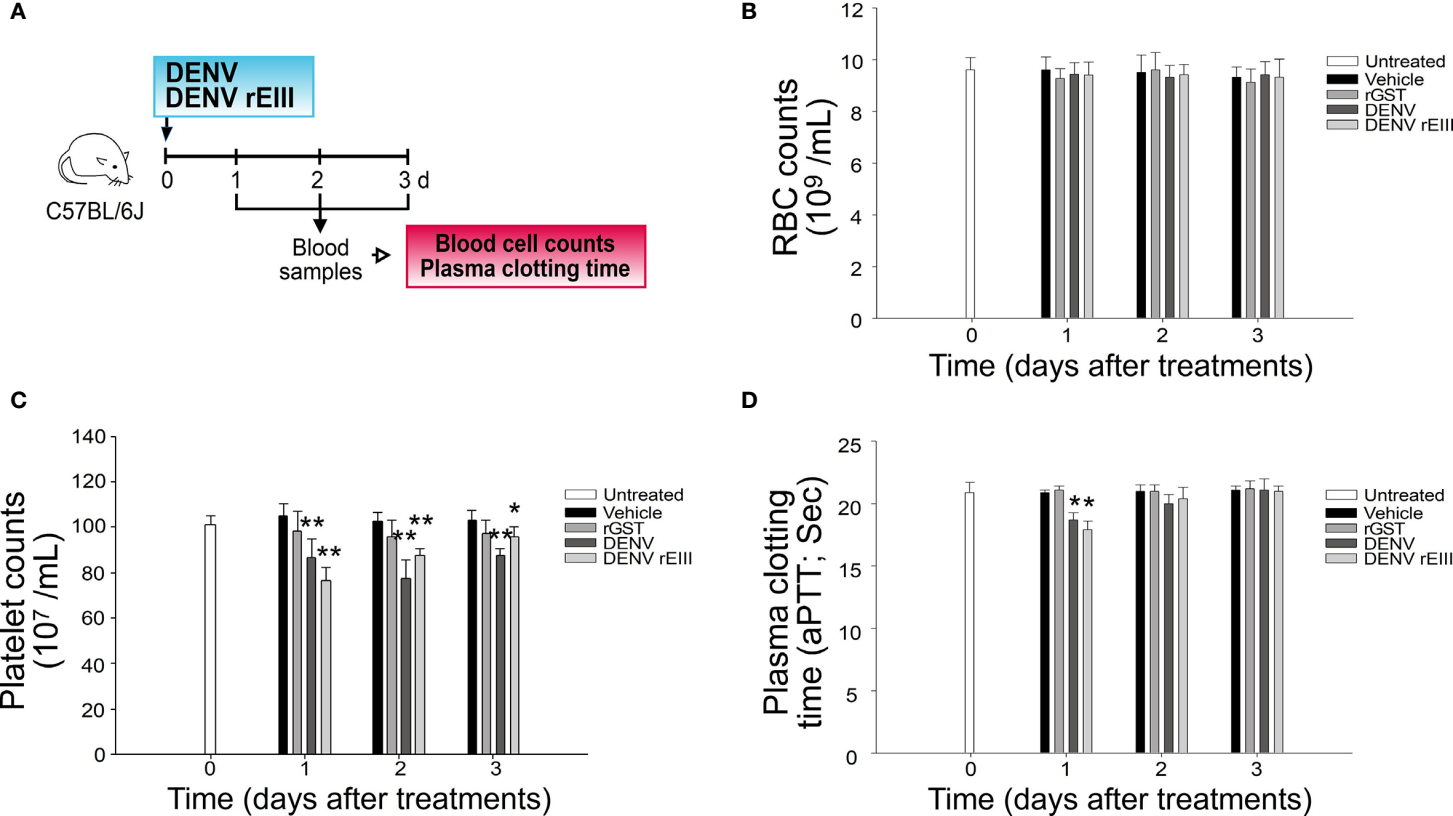
Challenges of rEIII-induced thrombocytopenia and hypercoagulation in mice. **(A)** Experiment outline. **(B)** RBC counts, **(C)** platelet counts, and **(D)** aPTT measures 1-3 d after mice were exposed to DENV particles (1.2 × 10^7^ PFU/kg; approximately 3 × 10^5^ PFU/25 g mouse) and rEIII (2 mg/kg). Compared with rGST treatments, injections of both DENV and rEIII induced **(C)** thrombocytopenia and **(D)** hypercoagulation (shortened aPTT plasma clotting time) in mice. *P < 0.05, **P < 0.01 *vs.* respective rGST groups; n = 6 (three experiments with two mice per group). The mouse drawing used in this and following figures was originally published in the *Blood* journal: Huang, H. S., Sun, D. S., Lien, T. S. and Chang, H. H. Dendritic cells modulate platelet activity in IVIg-mediated amelioration of ITP in mice. Blood, 2010; 116: 5002–5009. ^©^ the American Society of Hematology.

Soluble recombinant EIII (rEIII; **Figure S1**) can bind to and suppress the function of megakaryocytes (15). As the precursor cells of platelets, megakaryocytes express various platelet surface proteins (39). In this study, flow cytometry analysis results revealed that biotinylated DENV and various recombinant E fragments (domains I + II, II + III, and full length) could bind to platelets (**Figure 1** and **Figure S2**). Probably because of the protein folding structure, among all rE protein fragments, rEIII displayed the strongest platelet-binding property (**Figure 1**). The binding of both DENV and rEIII to platelets could be suppressed by neutralizing Igs against rEIII (**Figure S3**), indicating that rEIII and DENV share common and functional platelet-binding epitopes. Recombinant EIII bound to mouse platelets in a dose-dependent manner (**Figures S4A–D**). Compared with the negative control recombinant proteins (glutathione-*S* transferase and isotype control Ig), rEIII induced markedly higher levels of surface expression of P-selectin (**Figures S4E–H**, **S5**), which is a marker of platelet activation (18, 40). In addition, platelets preferentially bound to rEIII-coated but not rGST-coated cover slides, with higher percentages of activation-associated morphologies (**Figures S4I–P**). Mouse experiments further revealed that similar to challenges with DENV, injections of rEIII but not those of rGST induced thrombocytopenia and shortened plasma clotting time *in vivo* (**Figure 2**). These results suggest that exposure of DENV and virion-surface EIII to platelets and plasma is sufficient, leading to an abnormal coagulation response.

### Treatment With rEIII Abnormally Affect Plasma Clotting Time: Implications on Hemorrhage

**Figure 3:**
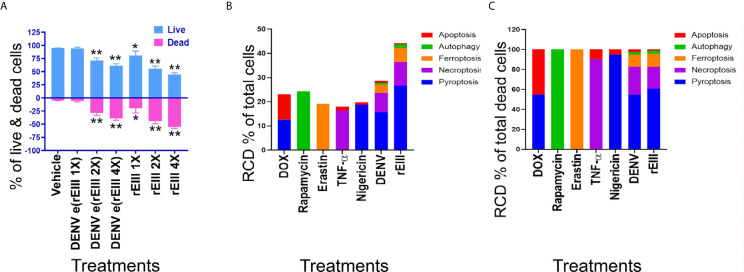
DENV- and rEIII-induced RCD in platelets. **(A)** Washed mouse platelets treated with vehicle and various doses of DENV and rEIII; the live and dead cell populations were estimated using Zombie-NIR Kit labeling and flow cytometry analysis. [rEIII: 1× = 0.3 μM, 2× = 0.6 μM, 4× = 1.2 μM; DENV e(rEIII 1×) is a DENV level equivalent to 0.3 μM rEIII, as indicated by the methods described in **Figure S8**]. **(B)** RCD inducers, doxorubicin (DOX; apoptosis) (2.5 μg/ml), rapamycin (autophagy) (0.5 μM), erastin (ferroptosis) (10 μM), TNF-α (necroptosis) (2.5 ng/ml), and nigericin (pyroptosis) (3.5 μM) induced relatively simple RCD patterns. By contrast, DENV and rEIII induced multiple RCD pathways, in which pyroptosis is the major RCD response, with counts approximately 60% of total RCD **(B)**. If we normalize the respective RCD by the population of death cells (dead cell population normalized to 100%), we can obtained a more similar RCD pattern in DENV and rEIII groups **(C)**. *P < 0.05, **P < 0.01 *vs.* vehicle groups.

In addition to platelets, rEIII can affect plasma clotting time because rEIII is a glycosaminoglycan (GAG)-binding protein. Experiments on aPTT revealed that because of the neutralizing effect on heparin, the addition of the heparin-binding proteins rEIII and protamine but not that of the control proteins, rGST and bovine serum albumin shortened heparinized aPTT plasma clotting time (**Figures S6A, B**). In addition to heparin, EIII binds to other GAGs (10), which may be used as antidotes to suppress any interaction between rEIII and heparin or the cell surface glycoprotein (10). Using this GAG binding property of EIII, here we developed aPTT-based assays for (I) screening selective rEIII-binding GAGs and (II) determining viremia equivalent doses of EIII. These two technologies (I and II) are useful for the EIII characterizations, while not relevant to the dengue pathogenesis. First, among the tested GAGs, we found that CSB most effectively blocked rEIII–heparin and rEIII–platelet binding (**Figures S6C, D**, **S7**). Second, based on this aPTT-based functional analysis, we measured and compared the heparin-binding properties of DENV and rEIII (**Figure S8**). For example, 0.3–1.2 μM of rEIII, cell-activating and cytotoxic dose for platelets (**Figures 3** **–** **6**) was estimated to be functionally equivalent to a virion titer within a previously published DHF viral load (41) range (**Figure S8**). Mouse experiments further revealed that CSB treatment not only inhibited rEIII–platelet binding *in vitro* but also ameliorated rEIII-induced thrombocytopenia and rEIII-prolonged bleeding time in mice (**Figure S6**).

### Pyroptosis Is a Major Cell Death Pathway of Platelets Treated With DENV and rEIII

**Figure 4:**
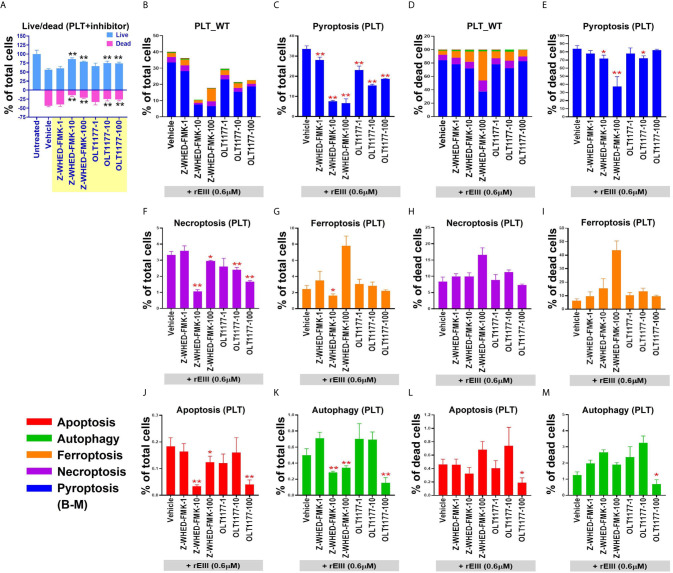
Treatment with Nlrp3-inflammasome inhibitors protects wild type (WT) mouse platelets (PLT) from DENV- (equivalent to 0.6 μM rEIII) and rEIII (0.6 μM)-induced pyroptosis. Treatment with Nlrp3 inhibitor OLT1177 (100 μM) and caspase 1 inhibitor Z-WHED-FMK (100 μM) rescued rEIII-induced platelet cell death **(A)**. Treatments with both OLT1177 and Z-WHED-FMK rescued % of RCDs **(B, C, F, G, J, K)**. Intriguingly, if we normalize the respective RCD % by the population of death cells (**D**; dead cell population normalized to 100%), we found that OLT1177 and Z-WHED-FMK preferentially rescued **(E)** pyroptosis, **(H, I, L, M)** but not the other tested RCDs. n = 6, *P < 0.05, **P < 0.01 *vs.* vehicle groups.

After the characterization of EIII-mediated effects on platelet binding and plasma clotting, we further investigated whether rEIII treatment is sufficient for inducing platelet cell death. Annexin V and caspase results suggested the involvement of platelet apoptosis in DENV-induced pathogenesis and its association with disease severity (42–44). Because annexin V and caspase signals can be detected in both apoptotic and non-apoptotic cell death (45–49), we investigated whether non-apoptotic, regulated cell death (RCD) pathways are also involved in DENV- and rEIII-induced platelet death. Accordingly, RCD pathways, including pyroptosis, necroptosis, ferroptosis, apoptosis, and autophagy were analyzed using flow cytometry analysis. Here we found that in agreement with platelet activation data (**Figures 1** and **S3**–**S5**), treatments with DENV and rEIII induced platelet cell death in a dose-dependent manner (**Figure 3A**). Various cell death inducers, including nigericin (pyroptosis), doxorubicin (apoptosis), rapamycin (autophagy), TNF-*α* (necroptosis), and erastin (ferroptosis) roughly induced the respective cell death pathway of tested platelets (**Figure 3B**, RCD % of total cells; 3C, RCD % of total dead cells; **Figure S9**, flow cytometry gating and calculations) (13, 14). Intriguingly, when compared with these cell-death agonists, treatments with DENV and rEIII induced considerable pyroptosis, necroptosis, and ferroptosis responses of platelets but only minor levels of apoptosis (**Figures 3B, C** **)**. In addition, cell death patterns were similar in DENV- and rEIII-treated groups in which pyroptosis displayed the highest levels in both groups among all tested RCD pathways (**Figure 3C**; >60%), suggesting that DENV-induced RCD is mediated through EIII moieties on virus particles. In addition, treatment with selective inhibitors of pyroptosis and necroptosis exerted ameliorative effects against DENV- and rEIII-induced platelet cell death (**Figure S10**) and further showed the involvement of these two major RCD pathways. Accordingly, the Nlrp3 inhibitor OLT1177 and the inflammasome/caspase1 inhibitor Z-WHED-FMK were used to further characterize whether Nlrp3 inflammasome is involved in such pyroptosis response. Analysis results indicated that both Nlrp3 (OLT1177) and inflammasome/caspase 1 inhibitor (Z-WHED-FMK) treatments reduced total platelet death (**Figures 4A, B** **)**, in which almost all types of RCDs seem to be suppressed (**Figures 4B, C, F, G, J, K**). While intriguingly, if we normalized various RCD cell population with the respective dead cell population (dead cell of each group normalized to 100%), (**Figures 4D, E, H, I, L, M**) we found that inhibitors of OLT1177 and Z-WHED-FMK preferentially suppressed pyroptosis (**Figure 4E** **)**, but not the other RCD pathways of platelets (**Figures 4H, I, L, M** **)**. To verify these pyroptosis results using different methods, we performed additional caspase-1 colorimetric assay, detections of cleaved GSDMD and cell-surface GSDMD levels, and the releases of IL-1β and LDH. Consistently, inflammasome inhibitors Z-WHED-FMK, OLT1177, and pyroptosis/GSDMD inhibitor DMF (14, 50) markedly rescued rEIII-induced pyroptosis and damages of platelets (**Figures S11**, **S12**). These results collectively suggest that EIII induced an Nlrp3 inflammasome-dependent pyroptosis in platelets.

In addition to EIII, soluble DENV NS1 levels also markedly increased in the circulation of DHF patients (51). Because DENV NS1 can activate inflammation through Toll-like receptor 4 (TLR4), NS1 is considered as one of the viral factors on the induction of dengue associated inflammation (52–54). Flow cytometry and platelet count analyses revealed that rEIII induced more pronounced platelet cell death, pyroptosis levels, and severer thrombocytopenia in mice, as compared to the rNS1 treatments (**Figure S13**). This suggests that, compared to NS1, EIII may be a more potent viral factor on the induction of platelet damage.

**Figure 5:**
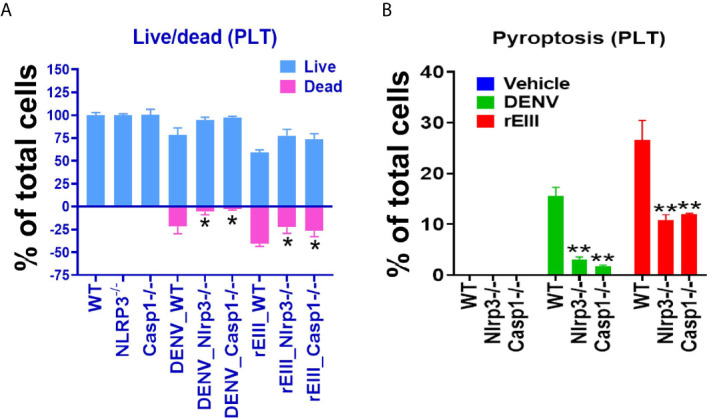
Nlrp3 and caspase-1 deficiencies protect platelets from DENV (equivalent to 0.6 μM rEIII) - and rEIII (0.6 μM)-induced RCD. **(A)** Compared with wild type (WT) controls, platelets from Nlrp3 (*Nlrp3^−/−^*) and caspase 1 (*Casp1^−/−^*) deficient mice displayed less cell death levels in response to DENV and rEIII exposures. **(B)** Similar to the cell death analysis, platelets from Nlrp3 (*Nlrp3^−/−^*) and caspase 1 (*Casp1^−/−^*) deficient mice displayed less pyroptosis levels in response to DENV and rEIII challenges. n = 6, *P < 0.05, **P < 0.01 *vs.* WT groups.

In addition to the use of Nlrp3 inhibitors, Nlrp3 and caspase 1 null mice were compared with wild-type mice. In agreement with inhibitor experiments, intravenous rEIII injections induced more pronounced platelet count reduction (**Figure S14**) and platelet activation (**Figure S15**) in wild type, compared to the Nlrp3 and caspase 1 mutant mice. Furthermore, we found that Nlrp3 and caspase 1 deficiencies protected mouse platelet from DENV- and rEIII-induced cell death (**Figure 5**). These results collectively suggested that Nlrp3 inflammasome is involved in DENV- and rEIII-induced platelet pyroptosis, and Nlrp3 inflammasome inhibitors display a protective role.

### Treatment With the Nlrp3 Inflammasome Inhibitor OLT1177 Ameliorate DENV- and rEIII-Induced Platelet Activation and Metabolic Burden of Mitochondria

**Figure 6:**
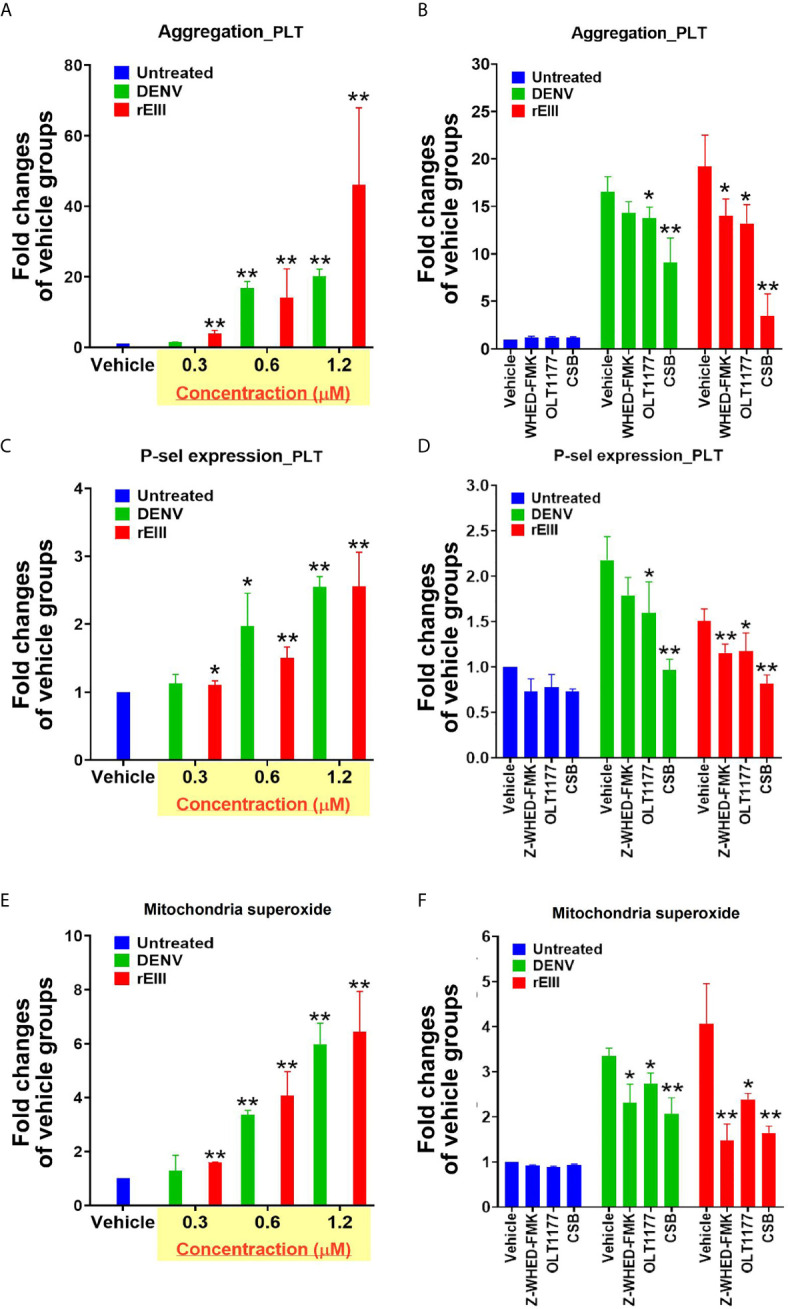
Nlrp3 and caspase-1 inhibitors protect platelets from DENV- and rEIII-induced activation. DENV- and rEIII-induced platelet activation, including **(A, B)** platelet aggregation, **(C, D)** platelet surface P-selectin expression, and **(E, F)** mitochondrial superoxide levels, could be suppressed with the treatments with Nlrp3 and caspase 1 inhibitors Z-WHED-FMK (10 μM) and OLT1177 (10 μM), respectively **(B, D, F)**. n = 6, *P < 0.05, **P < 0.01 *vs.* respective vehicle groups.

**Figure 7:**
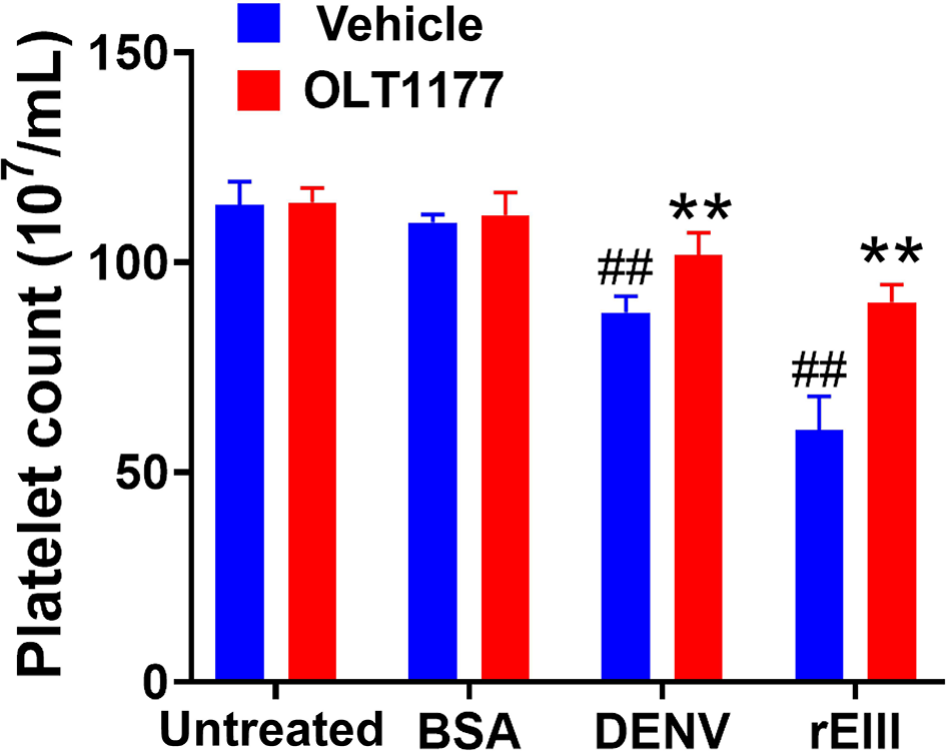
Nlrp3 inhibitor treatments ameliorate DENV- and rEIII-induced thrombocytopenia in mice. Treatments with Nlrp3 inhibitor OLT1177 (50 mg/kg) ameliorated DENV (3 × 10^5^ PFU/mouse; DHF viral load)- and rEIII (2 mg/kg)-induced thrombocytopenia in C57BL/6J mice. n = 6, ^##^ *P* < 0.01 *vs.* untreated groups; **P < 0.01 *vs.* respective vehicle groups.

Because Nlrp3 inflammasome-mediated pyroptosis is a major RCD involved in DENV- and rEIII-induced platelet defect, we further investigated whether the suppression of platelet Nlrp3 inflammasome through inhibitor treatment is sufficient to ameliorate DENV- and rEIII-induced abnormal platelet activation. Here we found that DENV and rEIII induced platelet aggregation, surface P-selectin expression, and increased mitochondrial superoxide levels in a dose-dependent manner (**Figures 6A, C, E**; gating **Figure S16**), whereas treatments with the Nlrp3 inflammasome inhibitors OLT1177 and Z-WHED-FMK ameliorated such abnormal platelet responses (**Figures 6B, D, F** **)**. Animal experiments further revealed that treatment with the Nlrp3 inflammasome inhibitor OLT1177 markedly ameliorated DENV- and rEIII-induced thrombocytopenia in wild-type mice (**Figure 7**). These results collectively suggested that EIII is a virulence factor that induces thrombocytopenia, and Nlrp3 inflammasome is a critical target for DENV and EIII to induce platelet defects.

## Discussion

According to the World Health Organization guidelines, thrombocytopenia, a commonly observed symptom in both DF and DHF (55, 56), is one of the crucial indicators of clinical severity (57). Mechanisms underlying thrombocytopenia and bleeding during DENV infection are not completely understood. Because patients with DF show thrombocytopenia and coagulant parameter changes (55, 56), DENV alone, without secondary infection-eliciting DHF pathogenesis, is sufficient to cause platelet and coagulation defects. Hypotheses have been proposed to explain the mechanism involved. For example, studies have suggested that the increased destruction of platelets through an ongoing coagulopathy process or increased peripheral sequestration causes thrombocytopenia in dengue (55, 56). However, the exact reasons and viral factors that lead to thrombocytopenia remain elusive. DENV EIII has shown to have cytotoxic effect on megakaryocytes (15, 58). At the same time, DENV NS1 was demonstrated to enhance endothelial permeability and vascular leaks through a toll-like receptor 4 (52, 53). Both DENV virus particle-associated EIII and soluble NS1 could be detected at high levels prior to the acute phase of DHF (51, 59) and may be considered as two virulence factors. Using clinically detected doses, here we found that intravenous treatments of both rEIII and rNS1 can activate platelets, induce platelet cell death and thrombocytopenia in mice. Despite these, compared to NS1, rEIII induced much higher levels of platelet pyroptosis and thrombocytopenia in mice (**Figure S13**). Because the induction of virion-surface EIII and soluble NS1 is induced in a similar, but not a same time course (51), the respective pathogenic role of EIII and NS1 on the elicitation of DHF-related pathogenesis remains to be further studied. However, data obtained in this present study suggested that virion-surface EIII is a candidate virulence factor that contributes to dengue-elicited thrombocytopenia. In addition, similar to soluble NS1, virion-surface EIII is a virulence factor that contributes to the first-hit in our two-hit DHF model (13, 16).

Primarily based on annexin V and caspase data, reports have suggested that platelet apoptosis is involved in DENV-induced pathogenesis and is associated with disease severity (42–44). However, because non-apoptotic cell death signaling pathways have recently been discovered, annexin V and caspase signals can be detected in various other types of RCD (45–49). Therefore, after the re-examination of RCD pathways, in this present study, we found that pyroptosis and necroptosis are the two major types of DENV- and rEIII-induced platelet RCDs, with approximately 55–60% and 20–25% RCD-marker (+) population (**Figure 3C**), respectively. Pyroptosis and necroptosis belong to necrotic RCD pathways (60, 61). Inflammasomes regulate and interact with various RCDs (62–65). DENV-induced Nlrp3 inflammasome activation and pyroptosis have been described in DENV-stimulated macrophages and monocytes (12, 66, 67). Although DENV-induced platelet pyroptosis has not been clearly characterized, Nlrp3 inflammasome-mediated inflammation has also been reported in DENV-stimulated platelets and DENV-induced thrombocytopenia and coagulopathy (12, 16, 68). These results suggested that although necrotic RCD of platelets need further characterization, Nlrp3 inflammasome-mediated inflammatory responses in other cell types have been recognized in dengue.

Even in one single cell type, cell population is still heterogeneous. This is likely the reason why previous reports have revealed that pathogen infections and cytotoxic agent treatments can lead to multiple types of RCDs simultaneously (69–75). For example, apoptosis, necroptosis, and pyroptosis participate simultaneously during retinitis (69). Photodynamic therapy can induce multiple RCD pathways, including apoptosis and autophagy (70). Cellular stress can activate both receptor-induced lysosomal-dependent and mitochondrial-mediated cell death pathways, which may lead to both programmed necrosis and apoptosis (71). Similarly, as DENV and EIII have been reported to have multiple cellular targets, it is not surprising to detect multiple RCDs after DENV and EIII challenges. In this present report, we found that DENV and EIII but not the other cell death inducers, induced a similar RCD pattern in platelets (**Figures 3B, C** **)**. Although further investigations are needed, this platelet cell-type-specific RCD patterns (CTS-RCDPs) (13, 14) may be also useful on the characterization of specific pathway inhibitors; as inflammasome inhibitors OLT1177 and Z-WHED-FMK preferentially blocked pyroptosis, but not ferroptosis, apoptosis, and autophagy (**Figure 4E**, *vs.* **Figures 4I, L, M** **)**.

Various putative cellular DENV cellular targets are expressed by platelets. For example, recent lines of evidence have suggested that CLEC5A mediates DENV-induced inflammation (67, 76), and glycoprotein Ib (GP1b*α*; CD42b) is involved in DENV infection (77). Both CLEC5A and CD42b are expressed by platelets (78, 79). Because CLEC5A involves cell death regulations (80) and interaction of platelet CD42b with von Willebrand factor induces platelet cell death (78), competition assay was performed to investigate whether recombinant CLEC5A and CD42b can block rEIII induced RCD. Results revealed that recombinant CD42b can block both rEIII-platelet binding, and rEIII-induced platelet cell death (**Figure S17**). In addition, similar to DENV and rEIII that induced platelet RCDs, pyroptosis is the major RCD of anti-CD42b antibody that induced platelet cell death (**Figure S17**). These results suggested that CD42b and CLEC5A are potential cellular targets of EIII on platelets and is worthy of further investigations.

Cellular stresses, including stimulation of danger-associated molecular patterns and pathogen-associated molecular patterns, can activate NLRP3 inflammasome to initiate pyroptosis and release the proinflammatory cytokines IL-1β and IL-18 (81). NLRP3 inflammasome inhibition prevents a wide range of diseases, including Alzheimer disease, metabolic diseases, and infectious diseases (64). In this study, treatment with selective inhibitors against Nlrp3 inflammasome displayed rescue effects on DENV- and rEIII-induced platelet cell death *in vitro* and thrombocytopenia in mice, suggesting that Nlrp3 inflammasome is a potential target for the therapeutic intervention of DENV-induced platelet defects. In addition, with further upstream blocking, treatment with an EIII-competitive inhibitor CSB displayed promising rescue effects. Our data suggest that DENV-E is a virulence factor that contributes to platelet-associated pathogenesis in dengue. This concept may be useful for developing therapeutic strategies to manage DENV-induced hemorrhage pathogenesis.

## Data Availability Statement

The original contributions presented in the study are included in the article/**Supplementary Material**. Further inquiries can be directed to the corresponding author.

## Author Contributions

H-HC conceptualized and supervised this project. T-SL, HC, D-SS, J-CW, Y-YL, and G-LL performed experiments and analyzed the data. H-HC wrote this manuscript. All authors contributed to the article and approved the submitted version.

## Funding

Ministry of Science and Technology, Taiwan (101-2320-B-320-004-MY3, 105-2923-B-320-001-MY3, 107-2311-B-320-002-MY3), Tzu-Chi University (TCIRP95002; TCIRP98001; TCIRP101001), and Tzu-Chi Medical Foundation (TC-NHRI105-02; TCMMP104-06; TCMMP108-04; TCAS-108-01).

## Conflict of Interest

The authors declare that the research was conducted in the absence of any commercial or financial relationships that could be construed as a potential conflict of interest.
