# Supplementary material for: Exposure of Platelets to Dengue Virus and Envelope Protein Domain III Induces Nlrp3 Inflammasome-Dependent Platelet Cell Death and Thrombocytopenia in Mice
Source: Front Immunol. 2021 Apr 29;12:616394. doi: 10.3389/fimmu.2021.616394 (PMC8118162; doi:10.3389/fimmu.2021.616394)
Supplement: Supplementary file 1 [file DataSheet_1.docx]

Supplementary Material

**Exposure of platelets to dengue virus and envelope protein domain III induces Nlrp3 inflammasome-dependent platelet cell death and thrombocytopenia in mice**

Te-Sheng Lien^†^, Hao Chan^†^, Der-Shan Sun, Jhen-Cheng Wu, You-Yen Lin, Guan-Ling Lin and Hsin-Hou Chang^*^

Department of Molecular Biology and Human Genetics, Tzu-Chi University, Hualien 970, Taiwan.

*** Correspondence:**Hsin-Hou Chang
hhchang@mail.tcu.edu.tw

† These authors share equals contribution

# Supplementary Figures S1-S9

**Figure S1**

**
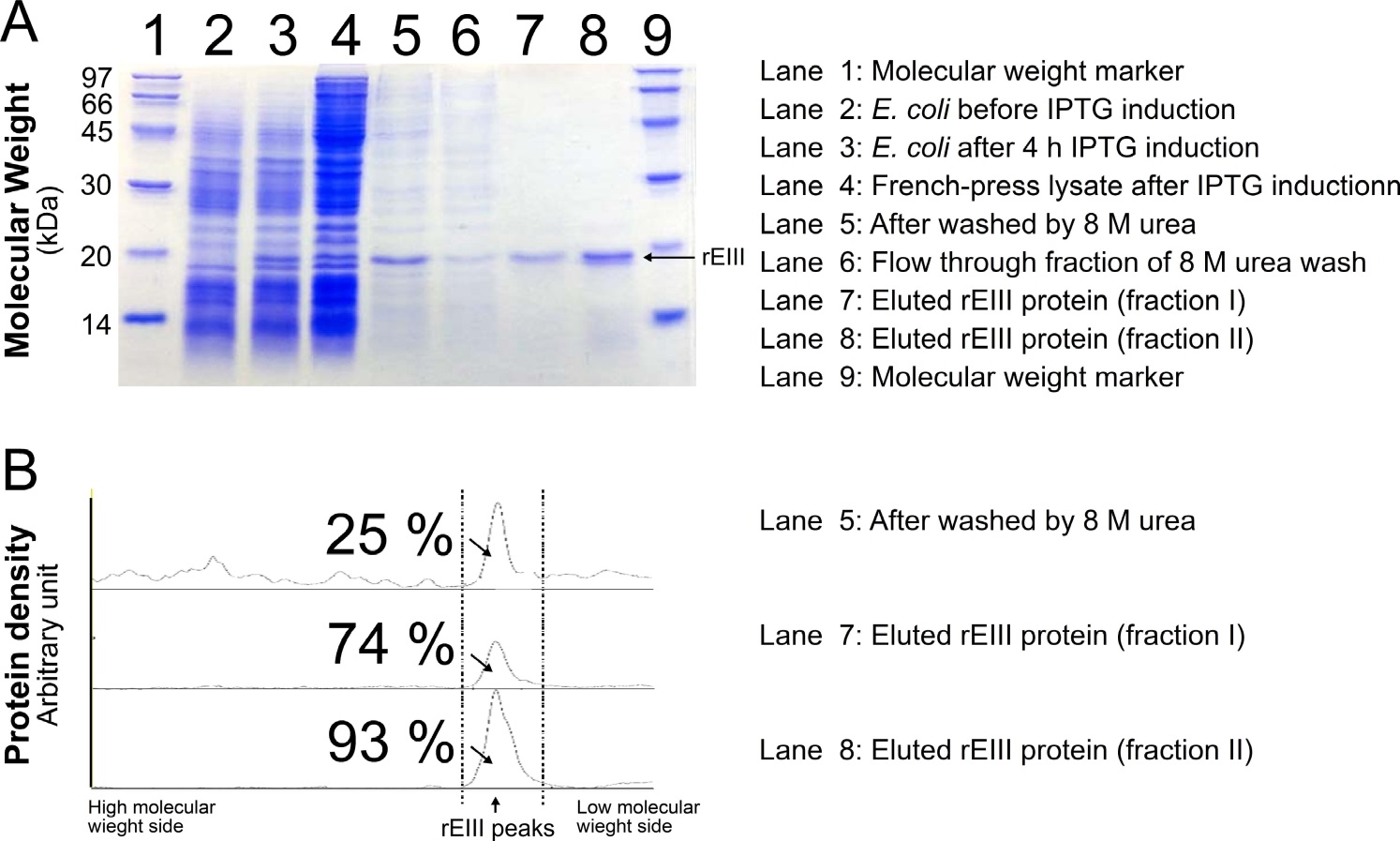
**

**Supplementary Figure 1. Analysis of recombinant DENV envelope protein domain III (rEIII).** The rEIII was overexpressed in *E. coli* BL21(DE3) after isopropyl β-D-1-thiogalactopyranoside (IPTG) induction. The protein fractions are separated by SDS-PAGE (A); purify of rEIII protein was characterized using ImageJ software (B). The purified rEIII with purify up to 90 % were used in the experiments.

**Figure S2**

**
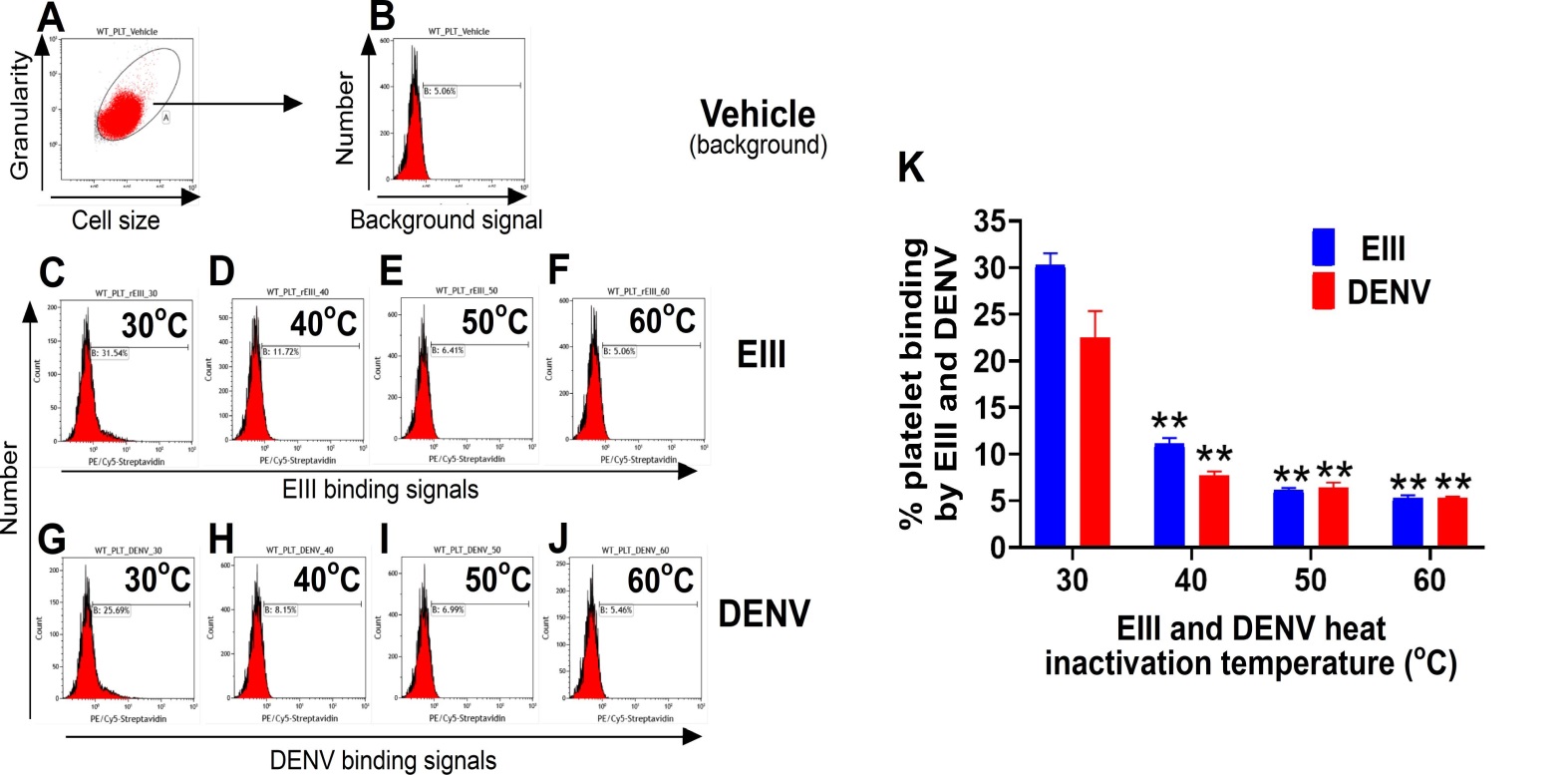
**

**Supplementary Figure 2. Platelet-binding assay for DENV particles and recombinant DENV envelope protein domain III (rEIII).** Flow cytometry analysis reveals the purified mouse platelet with gating using cell size and granularity (A), and background fluorescent signals (B). After placed at conditions with different temperature [30^o^C (C, G), 40^o^C (D, H), 50^o^C (E, I), 60^o^C (F, J)] for 30 min, DENV and rEIII were subjected to platelet binding for additional 30 min. The binding signals of biotinylated EIII (C-F) and DENV (G-J) to platelets were stained by fluorescence conjugated –streptavidin. Quantified results were showed (K). The 30^o^C groups were normalized to 100% (K). ** *P* < 0.01 vs. respective 30^o^C groups, n = 3. These results suggest that EIII is properly folded with comparable platelet-binding property as compared to DENV particles; and such platelet-binding property can be disrupted through high temperature (40^o^C-60^o^C) induce protein-misfolding.

**Figure S3**

**
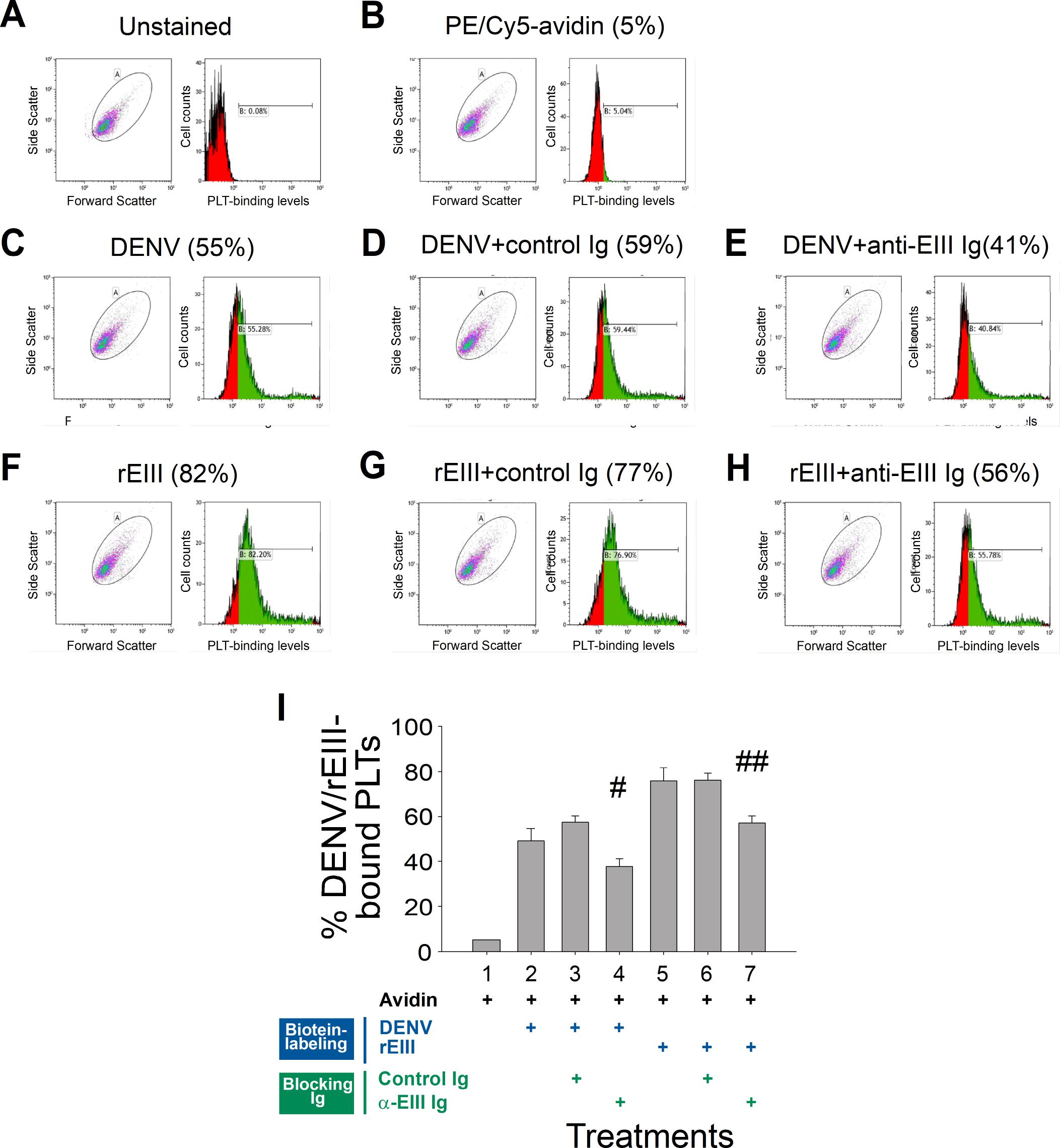
**

**Supplementary Figure 3. Blockage of DENV and rEIII binding to platelets by neutralizing antibody.** The blocking effect of anti-rEIII neutralizing antibody on biotinylated DENV-, and biotinylated rEIII-platelet binding was analyzed using flow cytometry; respective dot blots and histograms of unstained control (A), avidin (B), DENV (C), DENV + control Ig (D), DENV + anti-rEIII Ig (E), rEIII (F), rEIII + anti-rEIII Ig (G) and rEIII + anti-rEIII Ig (H) are indicated (A-H), and quantified (I). n = 6, # *P* < 0.05, ## *P* < 0.01 vs. respective control Ig groups.

**Figure S4**


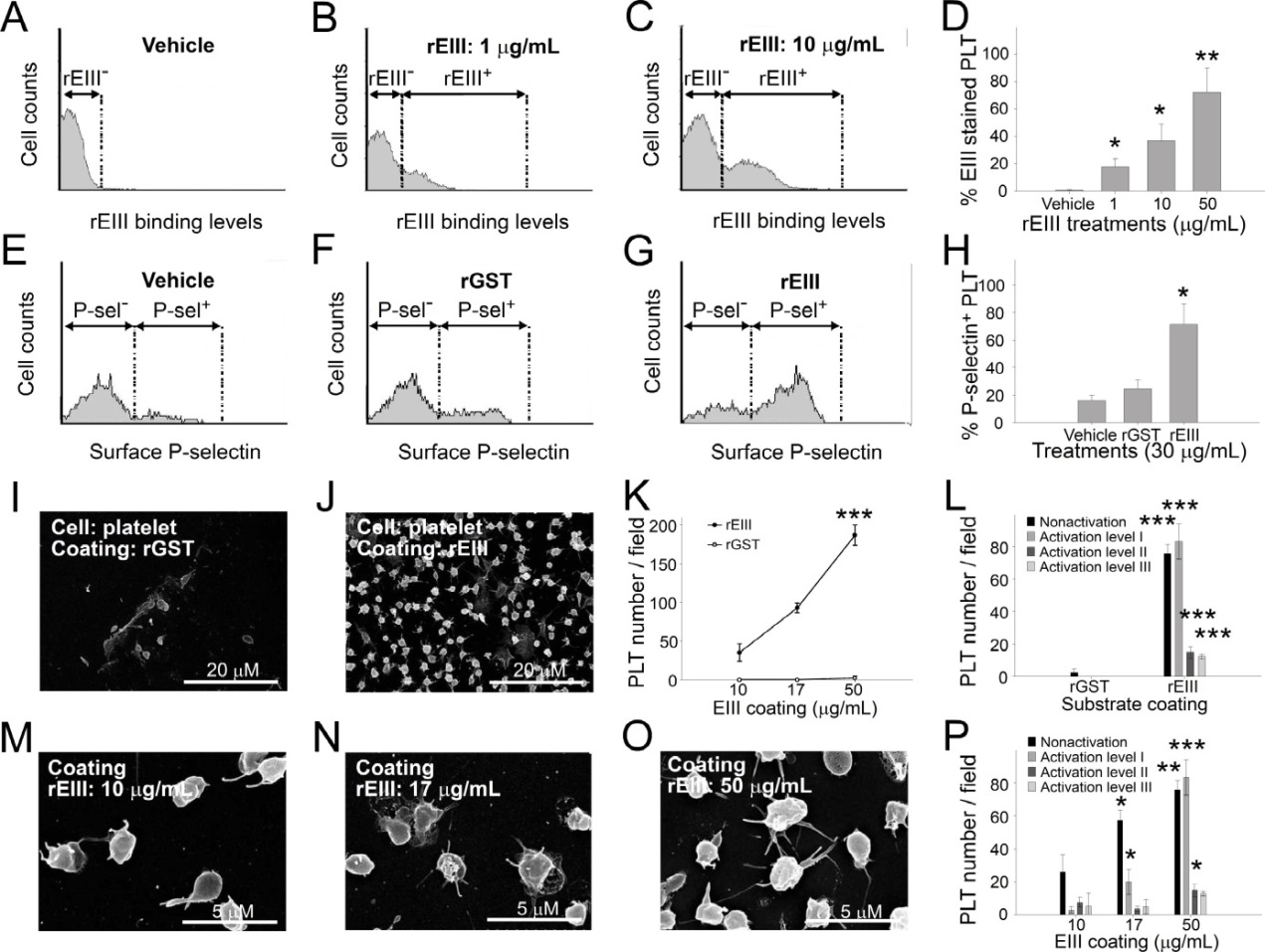


**Supplementary Figure 4. Soluble recombinant dengue virus envelope protein domain III (rEIII)-induced platelet activation.** The rEIII bound to (A-D) and activated (E-H) platelets (PLTs) in a dose dependent manner (D, H). Recombinant GST (rGST) was used as a control protein for comparisons (F, H). The rEIII-coated cover slides induced adherent platelets displaying activation morphology compare to rGST (I-L), and in a dose dependent manner (M-P). * *P* < 0.05, ** *P* < 0.01, *** *P* < 0.001, vs. respective vehicle groups in D; vs. respective rGST groups in H, K and L; vs. 10 μg/mL rEIII groups in P (n = 6; 3 experiments with 2 replicates).

**Figure S5.**

**
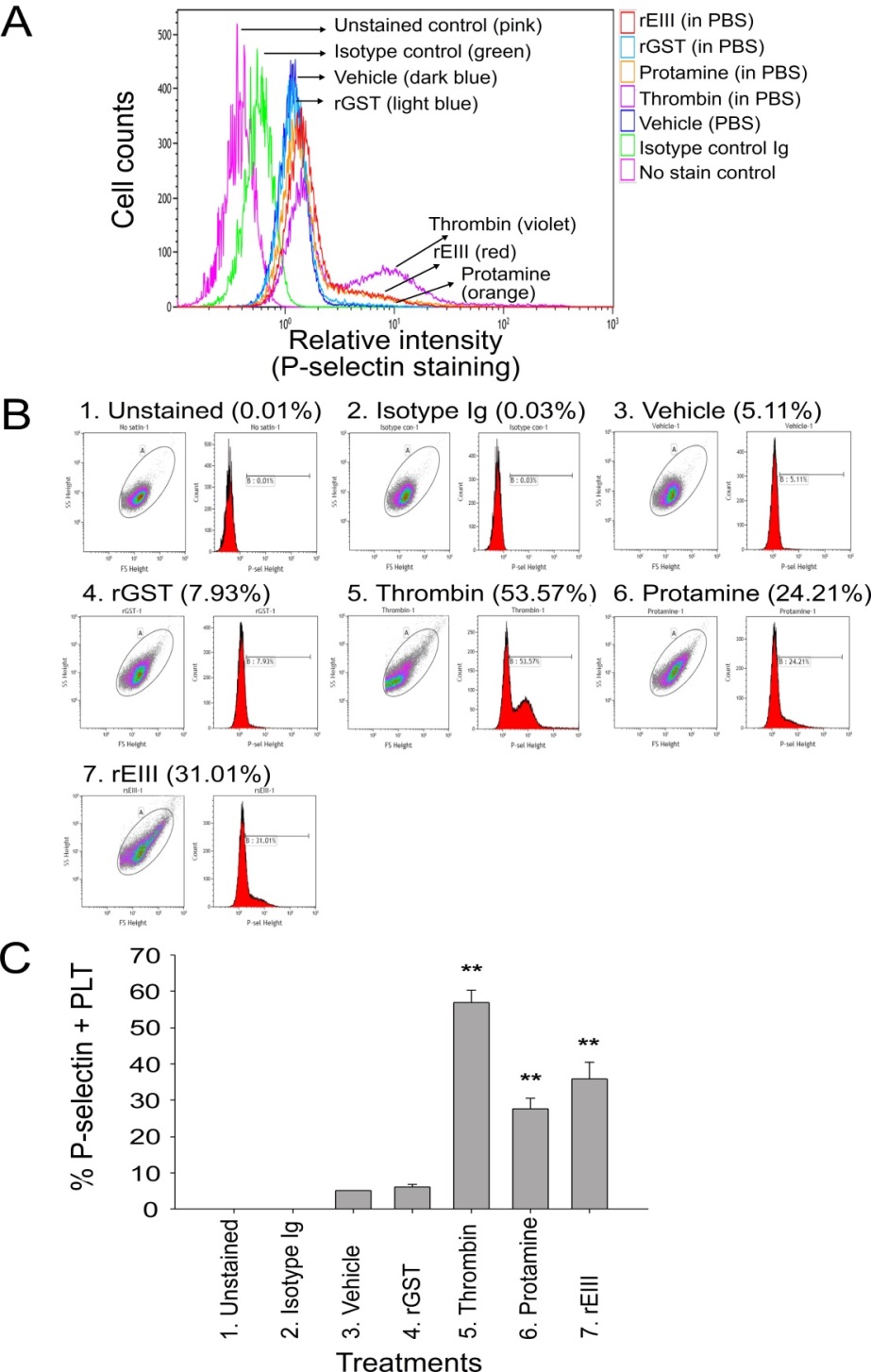
**

**Supplementary Figure 5.** **Flow cytometry analysis of rEIII-induced platelet surface P-selectin expression.** Overlayed (A) and respective (B) flow cytometry histograms of surface P-selectin expression levels of mouse platelets (PLTs) were stained with mouse anti-mouse P-selectin monoclonal Ig (IgG1-phycoerythrin PE-labeling, eBioscience) after treatments of vehicle (phosphate buffered saline, PBS, pH 7.4; dark blue), rGST (30 μg/mL; light blue), rEIII (30 μg/mL; red), protamine (30 μg/mL; orange) and thrombin (1 U/mL; violet) for 30 min. The fluorescence intensities in the “unstained (pink)” and “isotype Ig (green)” controls are also indicated. Protamine, a clinical-used antidote for heparin overdosing, was used as a positive control protein for heparin-binding. The quantified results were showed in C; n = 6, ** *P* < 0.01 vs. vehicle groups. Protamine used as a positive control of heparin- and glycosaminoglycan-binding protein (vs. rEIII).

**Figure S6**


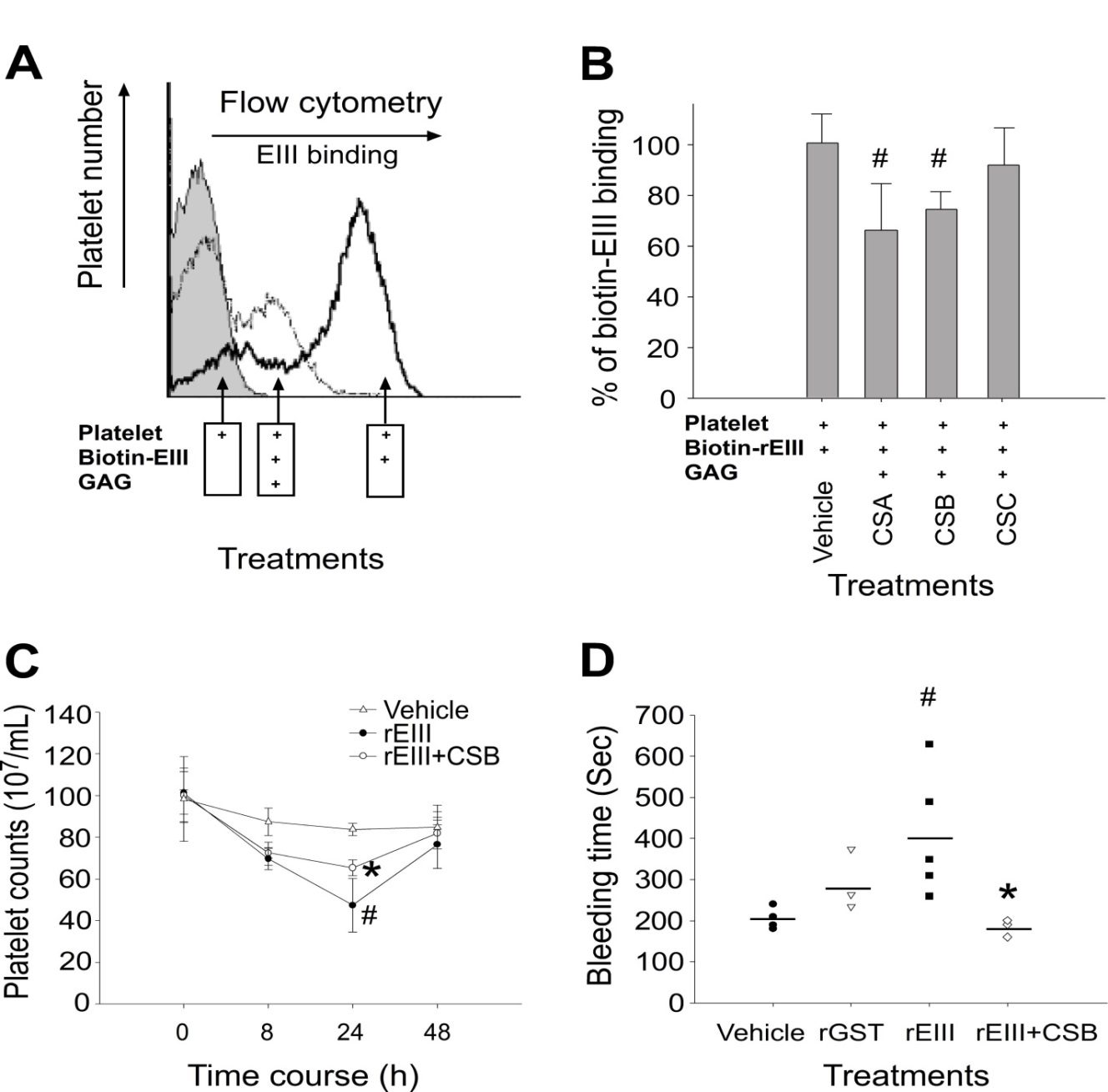


**Supplementary Figure 6.** **Treatment of chondroitin sulfate B (CSB) blocks platelet-rEIII binding**. Chondroitin sulfates (CSA, CSB, and CSC; 10 μg/mL) can serve as competitors against rEIII-heparin (0.3 μM) binding; flow cytometry was therefore further used to analyze the competition properties of CSA, CSB and CSC against rEIII-platelet interaction *in vitro* (A). Analysis results suggested that CSA and CSB exert higher competing properties compare to CSC (B). Because CSB exerts highest competing property against rEIII-heparin binding (Fig. S7F), CSB was used in the following *in vivo* analyses. Platelet counts and bleeding time analyses both indicated that the treatments of CSB (0.5 mg/kg) markedly ameliorated rEIII-challenge induced platelet counts reduction (C) and prolonged bleeding time (D) in mice. # *P* < 0.05 vs vehicle groups; * *P* < 0.05 vs rEIII groups; n = 6 (three experiments with 2 replicates) in (B); n = 5 (rEIII), n = 4 (vehicle, rGST, rEIII+CSB) (2 experiments with 2 or 3 replicates) in (C, D).

**Figure S7**


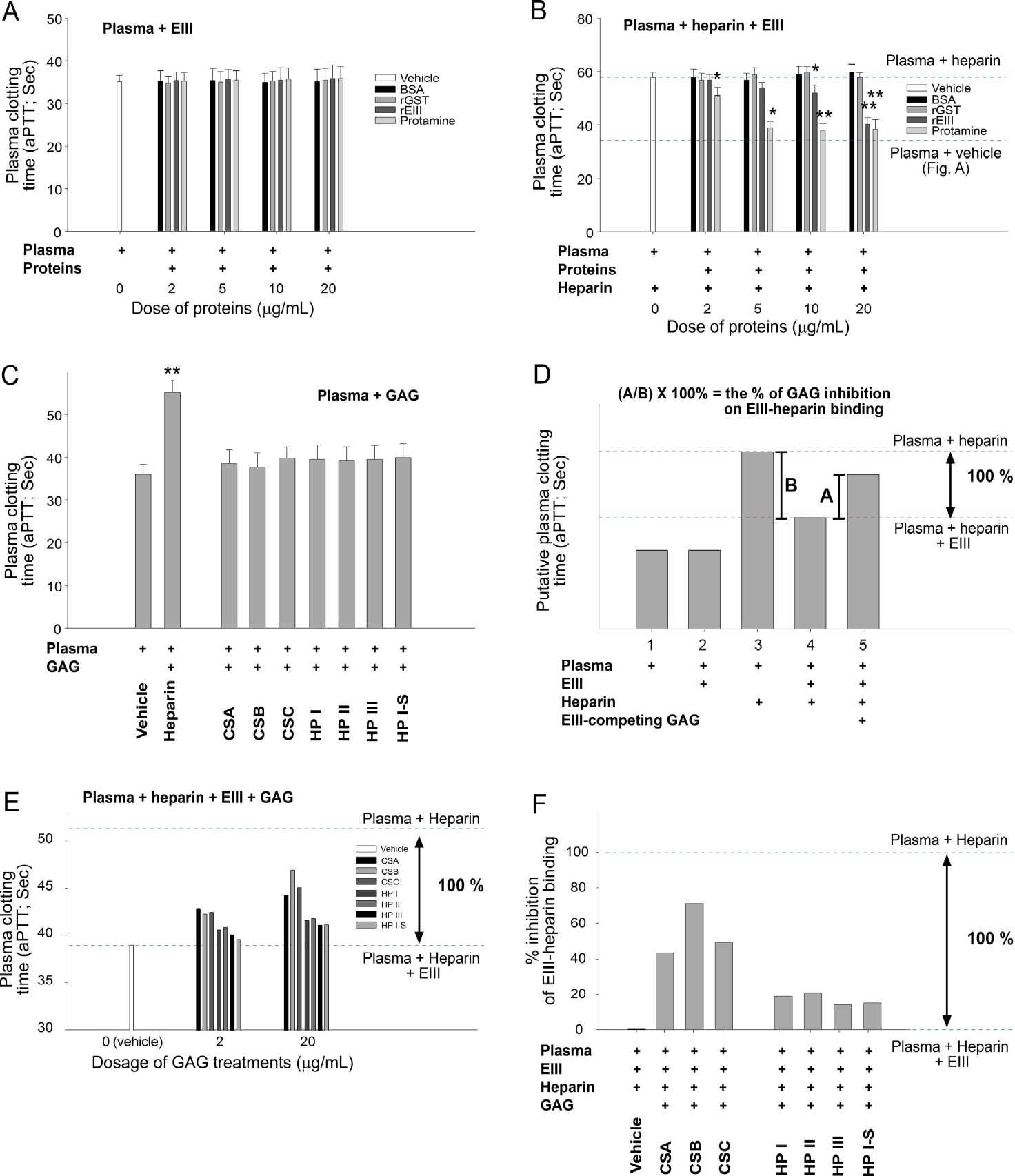


**Supplementary Figure 7.** **Characterization of relative EIII-binding properties of glycosaminoglycans (GAGs) through analyzing aPTT using heparinized rabbit plasma**. Addition of rEIII (2-20 μg/mL) or protamine (2-20 μg/mL) alone to the rabbit plasma did not influence aPTT (A); however, same treatments of rEIII (10-20 μg/mL) and protamine (2-20 μg/mL) drastically rescued heparin prolonged aPTT (B). Protamine is an US-FDA approved antidote for heparin, and used as a positive control of heparin binding protein (A, B). Theoretically, supplement with additional GAGs without anticoagulant properties (C) to compete rEIII-heparin binding can block rEIII induced clotting time shortening effect of heparinized plasma (D). The efficiency of GAGs on the rescue of heparin prolonged aPTT was calculated by following formula: [A (GAG-prolonged clotting time of rEIII and heparin containing plasma) / B (heparin-prolonged clotting time of heparinized plasma without rEIII)] × 100% (D). The GAG-prolonged clotting time of rEIII and heparin containing plasma was analyzed using GAGs including chondroitin sulfate A (CSA), CSB, CSC, heparin disaccharide I-H (He I), heparin disaccharide II-H (He II), heparin disaccharide IIII-H (He III), heparin disaccharide I-S (He I-S) (E). The respective blocking efficiencies/percentages of rEIII-heparin binding were analyzed and obtained (F). Among these tested GAGs, CSB exerts superior heparin competing property (F) (data analyzed using 20 μg/mL GAGs). * *P* < 0.05, ** *P* < 0.01 vs. respective vehicle groups. n = 6 (3 experiments with 2 replicates).

**Figure S8**


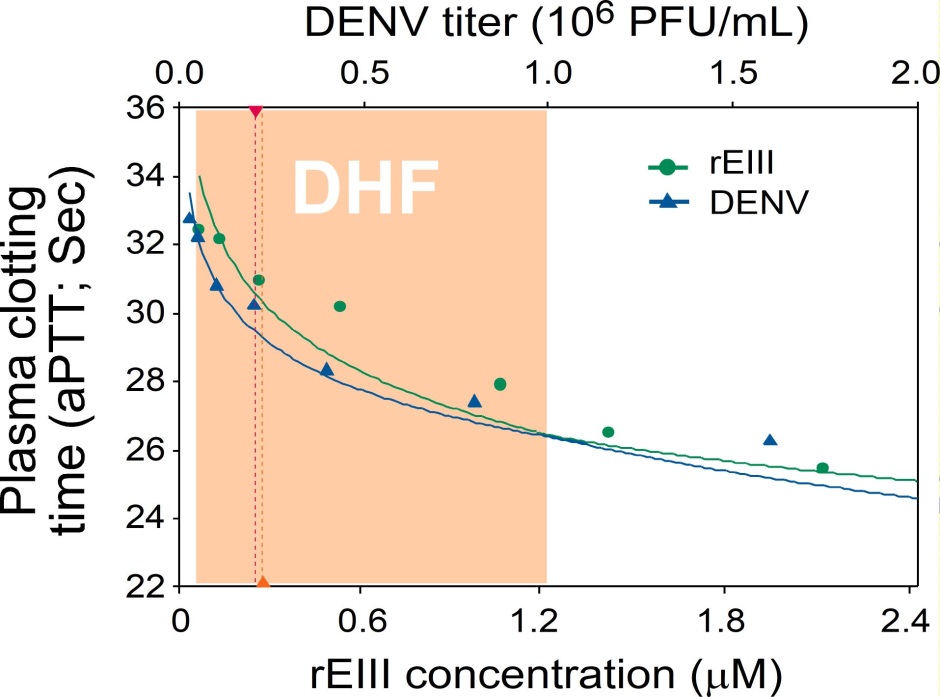


**Supplementary Figure 8.** **Analysis of functionally comparable and equivalent DENV and rEIII levels using aPTT plasma clotting time assay.** rEIII can suppress the anticoagulant property of heparin. To investigate the dose dependent response of DENV versus rEIII on plasma clotting using heparinized mouse plasma, regression curves were blotted by DENV and rEIII dosages versus aPTT. Accordingly, we found that the doses of DENV and rEIII treatments, in a similar way, both inversely correlated with the aPTT. Using this approach, we can determine functionally equivalent dosages of DENV and rEIII on the binding of heparin. For example, the estimated DHF viral load virion titer (1 × 10^3^ ~1 × 10^6^ PFU) is approximately equivalent to 1.7 × 10^-3^~1.2 μM rEIII (flesh color labeled region). The dose 0.3-1.2 μM of rEIII that used in this report for the platelet analyses is located within this range.

**Figure S9**

**
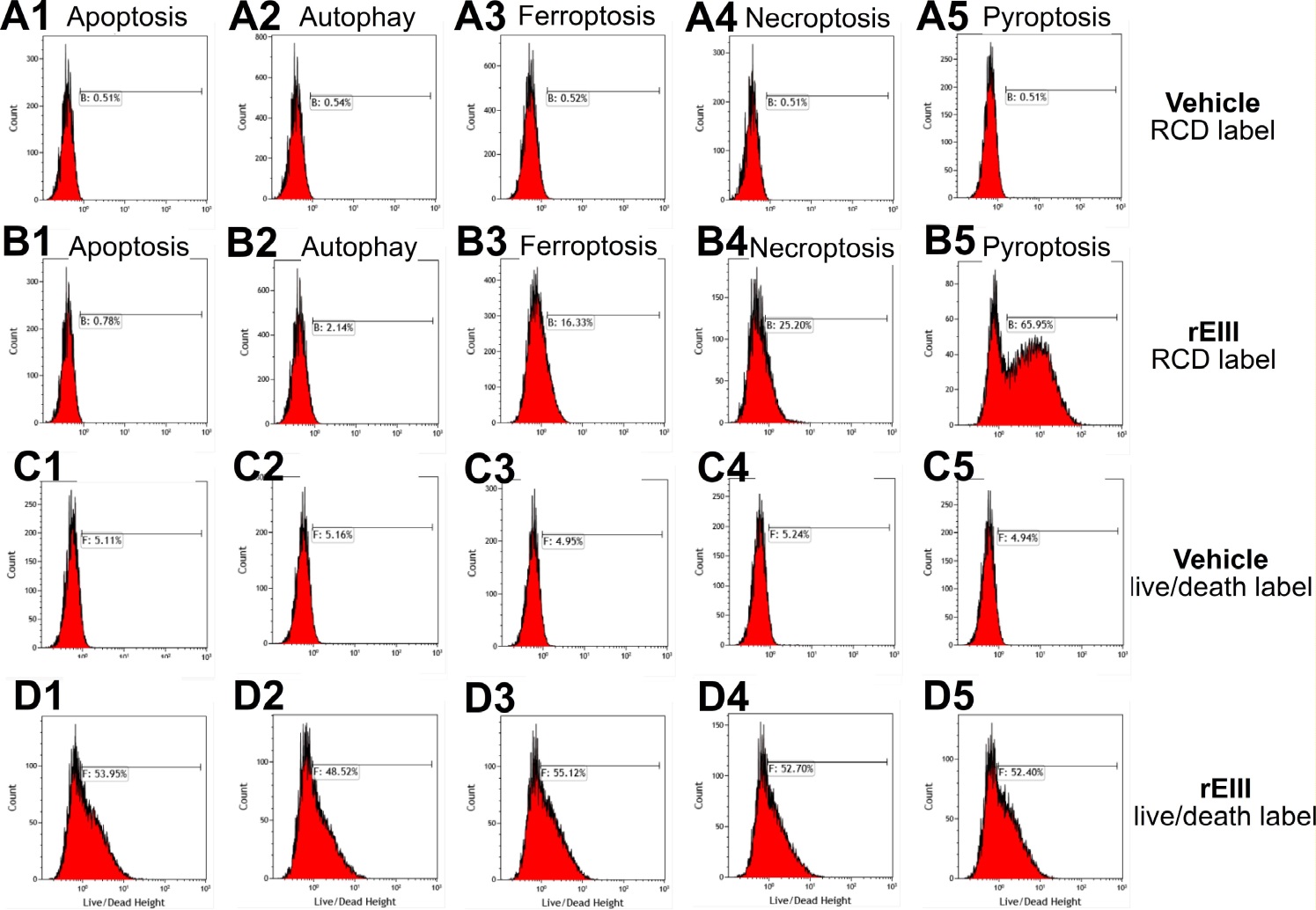
**

**Supplementary Figure 9.** **Calculation of respective percentage of regulated cell death (RCD).** An example shows how respective RCD pathway percentages were determining by flow cytometry results. Because overlapping of detection wavelengths, it is not feasible to detect total 5 RCD pathways simultaneously in one cell-staining sample. Therefore, we performed double staining of respective RCD plus cell live and death statue staining (an internal control). For example, after platelet samples were treated with vehicle (A, C) or rEIII (B, D), these platelets were subjected to respective RCD (A, B) and cell-live/death status (C, D) staining. The respective increased cell death signal (e.g. apoptosis signal B1-A1; △apoptosis) was normalized (e.g. B1-A1/D1-C1; △ apoptosis/△total death cell) by increased death-cell population (e.g. D1-C1; △total death cell) of respective RCD staining. The sum of B1-A1/D1-C1, B2-A2/D2-C2, B3-A3/D3-C3, B4-A4/D4-C4 and B5-A5/D5-C5 was considered as 100%. As a result, the pyroptosis % was calculated as [B5-A5/D5-C5] / [(B1-A1/D1-C1) + (B2-A2/D2-C2) + (B3-A3/D3-C3) + (B4-A4/D4-C4) + (B5-A5/D5-C5)] × 100%, and is approximately 60%. Results showed in the figure 3 were obtained through this formula using averaged results from triplicated samples of each group.

**Figure S10**

**
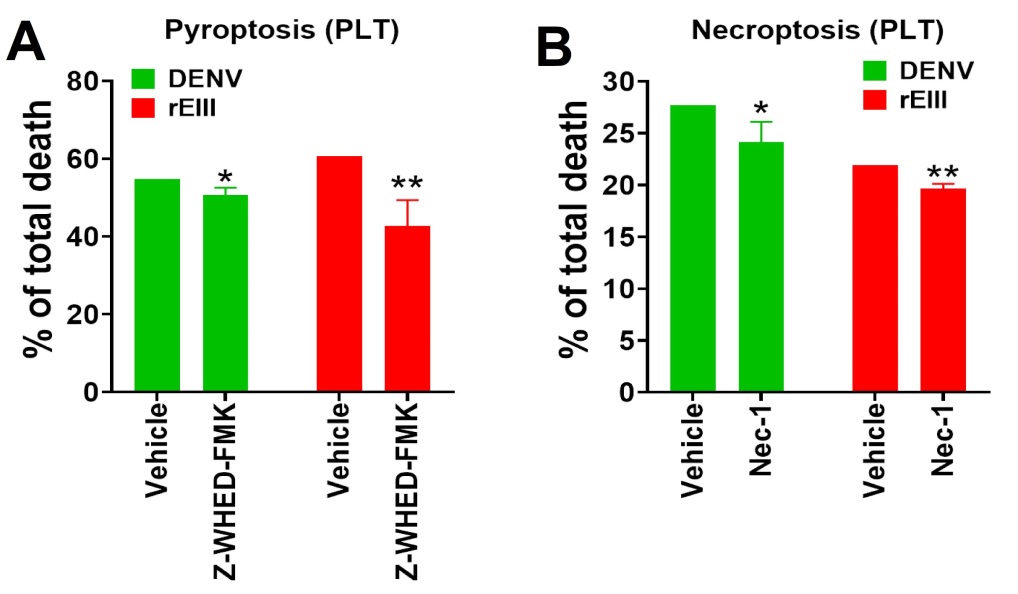
**

**Supplementary Figure 10.** **Rescue of DENV- and rEIII-induced platelet pyroptosis and necroptosis by selective inhibitors.** DENV- and rEIII-induced platelet pyroptosis and necroptosis can be rescued by selective inhibitors against pyroptosis (Z-WHED-FMK) (10 μM) and necroptosis (Nec-1) (50 μM), respectively. n = 6 (3 independent experiments with duplicated analyses). * *P* < 0.05, ** *P* < 0.01, vs. respective vehicle groups.

**Figure S11**

**
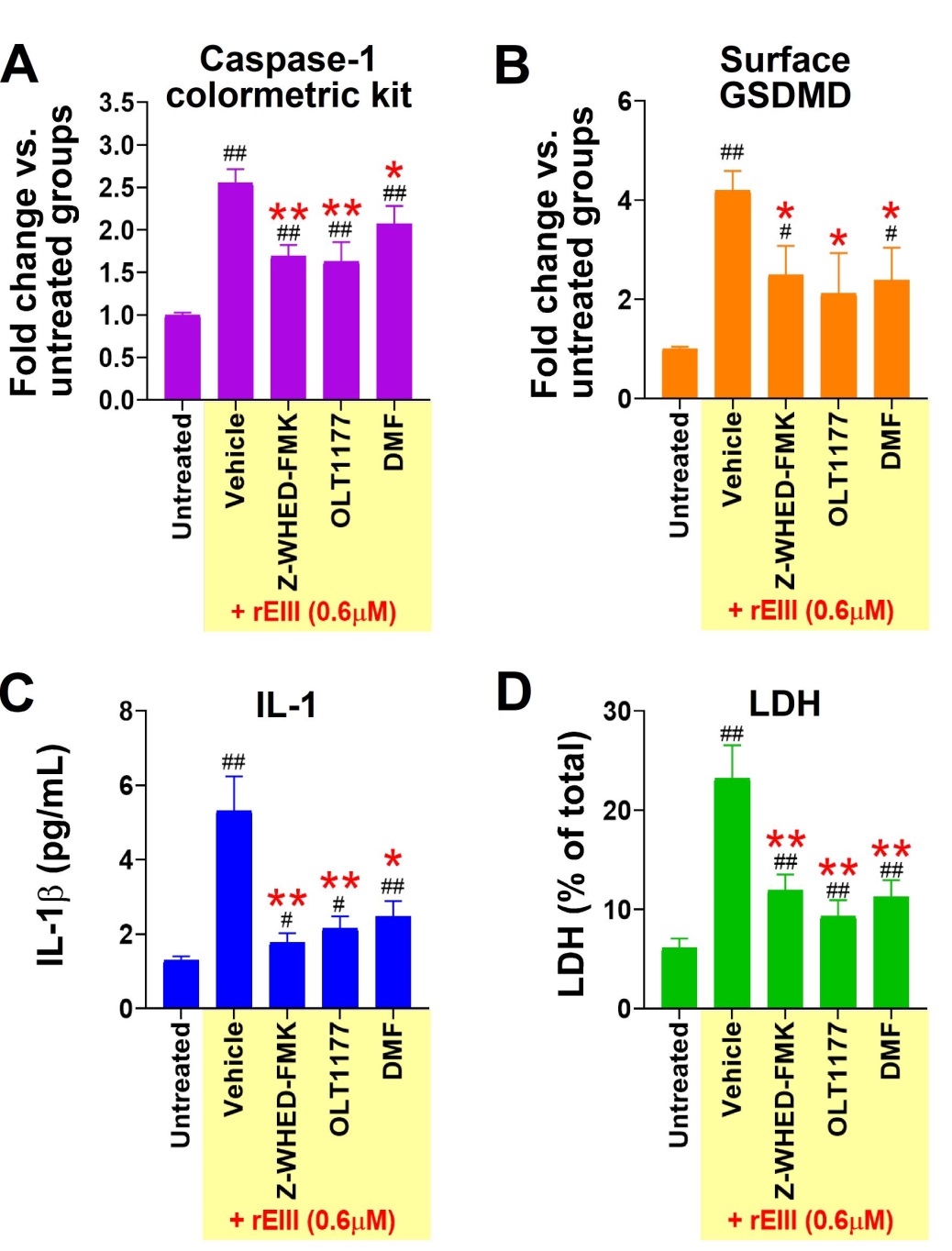
**

**Supplementary Figure 11.** **Rescue of rEIII-induced cell death by pyroptosis inhibitors.** Rescue of EIII (0.6 μM)-induced mouse neutrophil caspase-1 activation (A), cell-surface translocation of GSDMD (flow cytometry, without cell-membrane permeation) (B), IL-1β release (C), and lactate dehydrogenase (LDH) release (D), by 1 h respective pretreatments with pyroptosis inhibitors Z-WHED-FMK (10 μM), OLT1177 (10 μM), DMF (50 μM). The percentage % of LDH release is determined by following measurements: [released LDH/total LDH in lysis buffer (LDH kit, Abcam)-treated cell lysate] ×100%. n = 6, # *P* < 0.05, ## *P* < 0.01 vs. respective untreated groups; * *P* < 0.05, ** *P* < 0.01 vs. respective rEIII + vehicle groups.

**Figure S12**

**
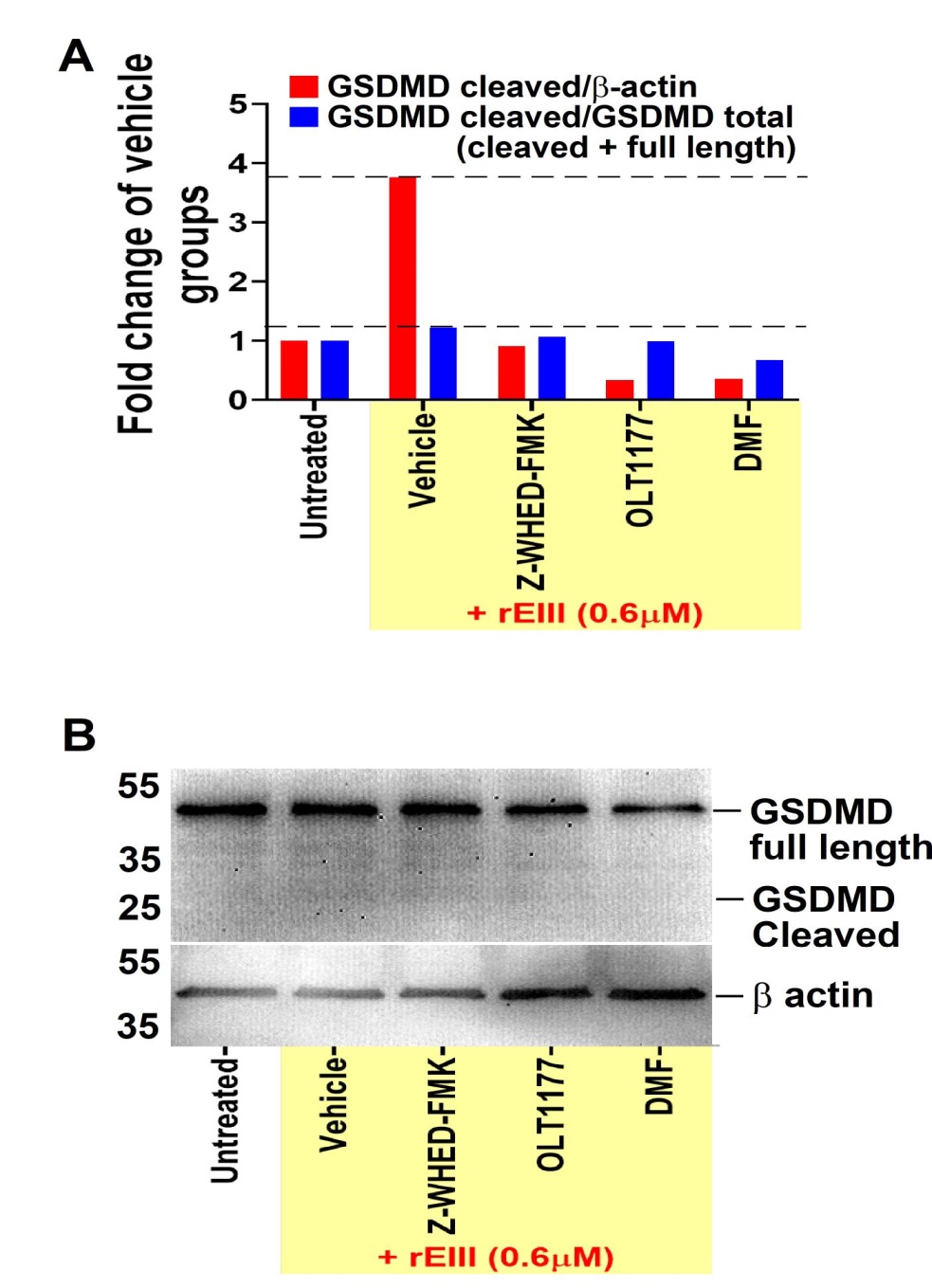
**

**Supplementary Figure 12.** **Western blotting analysis of platelet gasdermin D.** After treatments of rEIII and inflammasome inhibitors Z-WHED-FMK, OLT1177, dimethyl fumarate DMF (A, quantified results; B, blot images), cellular levels of full length and cleaved gasdermin D (GSDMD; a caspase-1 substrate and an essential pyroptosis mediator) of mouse platelets were analyzed by Western blotting analysis. Actin blots were used as internal loading controls. The blot-signal ratio of cleaved GSDMD/β-actin and GSDMD cleaved/total (cleaved + full length) in the untreated groups were normalized to 1 (A). After rEIII treatments, the ratios of GSDMD/β-actin, GSDMD cleaved/total, are increased (dashed lines); and such induction of cleaved GSDMD is generally suppressed by treatments of pyroptosis inhibitors z-WHED, OLT1177 and DMF (A).

**Figure S13**

**
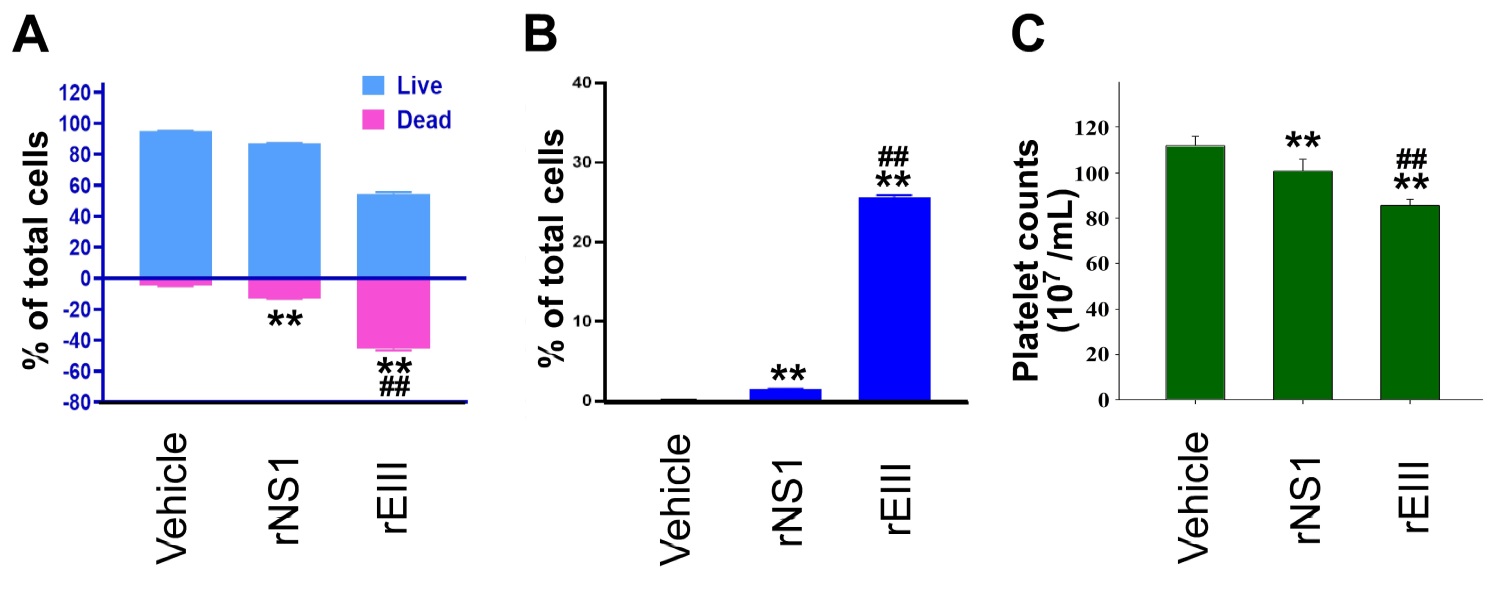
**

**Supplementary Figure 13. Treatments of DENV rNS1 and rEIII induced platelets cell death (A, B) *in vitro*, and reduced platelet counts (C) in mouse.** Flow cytometry analysis on the percentage of DENV rNS1and rEIII-induced cell death (A) and pyroptosis (B) in total analyzed platelet population. Dosage used rNS1, 0.6 μM, rEIII, 0.6 μM, both are adjusted approximately reached to the peak clinically detected levels. Following previously described methods, platelet counts of mice were analyzed 24 h after intravenous treatments of vehicle, rNS1 and rEIII (C). n = 6 (3 independent experiments with duplicated analyses). ** *P* < 0.01, vs. respective vehicle groups; ## *P* < 0.01, vs. respective rNS1 groups.

**Figure S14**

**
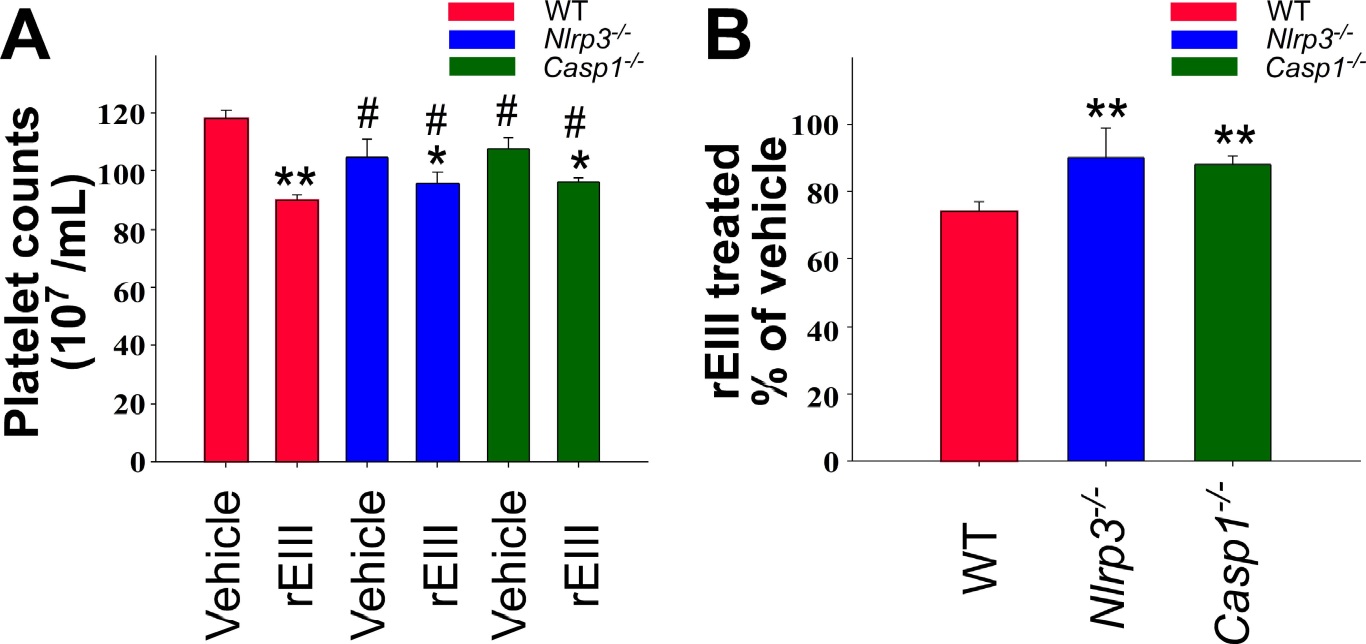
**

**Supplementary Figure 14. Mouse platelet counts.** After treated with vehicle and rEIII, platelet counts of wild type (WT), *Nlrp3^-/-^*, *Casp1^-/-^* gene knockout (KO) mice were recorded (A), and the % of platelet counts in the rEIII groups versus vehicle groups were also indicated (B). n = 6 (3 independent experiments with duplicated analyses). * *P* < 0.05, ** *P* < 0.01, vs. respective vehicle groups; # *P* < 0.05, vs. WT vehicle group (A); ** *P* < 0.01, vs. WT group (B).

**Figure S15**

**
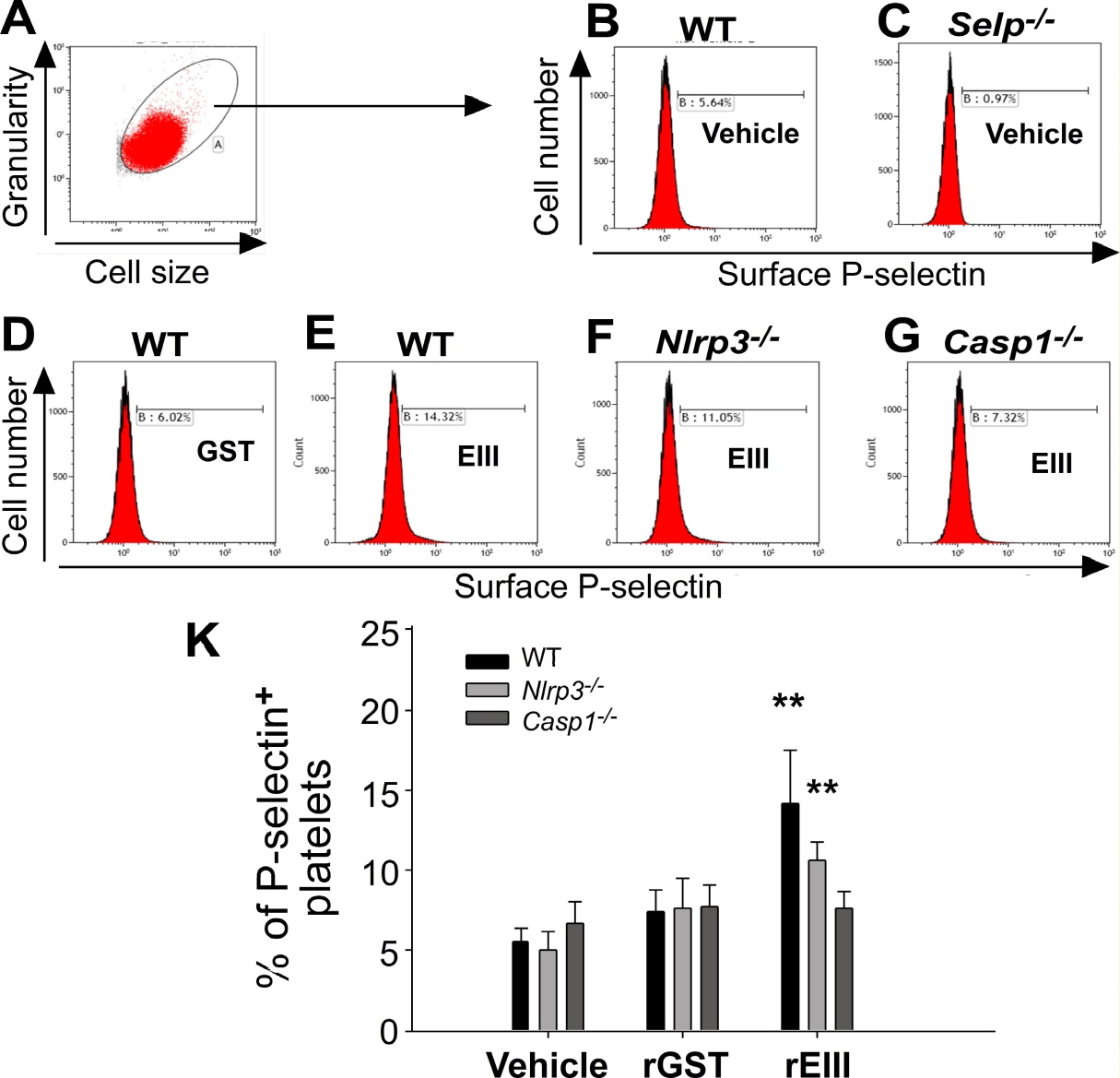
**

**Supplementary Figure 15. Treatments of rEIII induce platelet activation in mice.** Platelet activation *in vivo* was analyzed by surface P-selectin expression. Example images of flow cytometry gating (A), and the surface P-selectin expression of wild type (WT), *Nlrp3^-/-^*, *Casp1^-/-^* gene knockout (KO) mice after vehicle, rGST and rEIII (2 mg/kg) treatments were illustrated (B-G). The quantified results of platelet surface P-selectin expression were also indicated (K). n = 6 (3 independent experiments with duplicated analyses). ** *P* < 0.01, vs. respective vehicle and rGST groups.

**Figure S16**

**
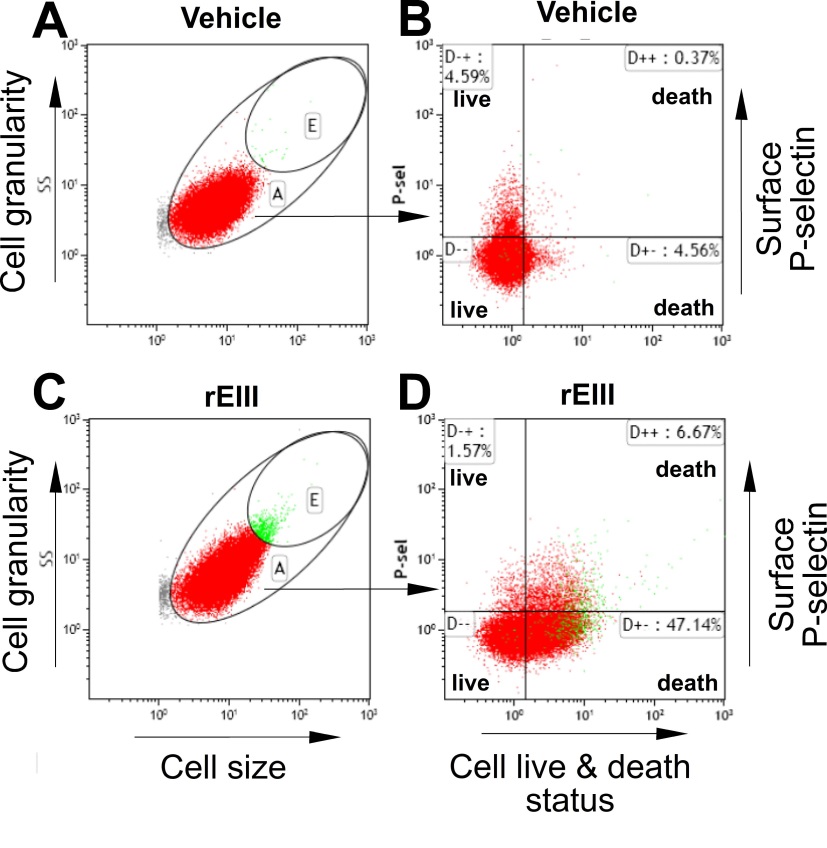
**

**Supplementary Figure 16.** **An example of flow-cytometry gating in the platelet activation and cell death analyses.** Vehicle (A, B) and rEIII (0.6 μM) (C, D) treated platelets were analyzed by flow cytometry. The area E, displaying higher levels of cell size and granularity, was considered as an aggregated population of platelets (A, C). The area A was further analyzed using specific reagent labeling, by which, the activated (surface P-selectin^+^) and death populations of platelet are revealed (B, D). Using a similar protocol, the detection for surface P-selectin staining antibody can be replaced by MitoSOX dyes for detecting the mitochondrial superoxide levels (Fig. 6).

**Figure S17**

**
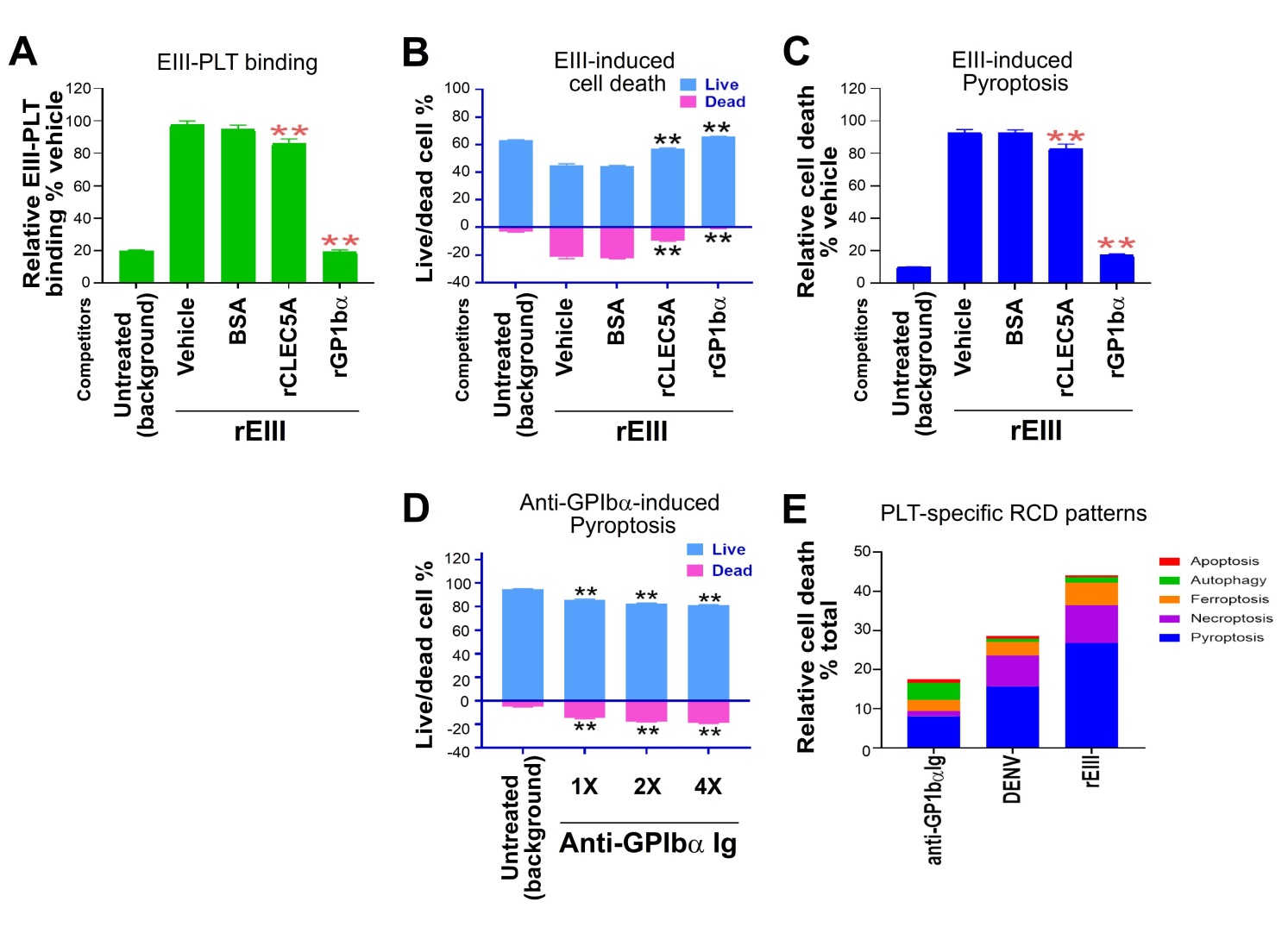
**

**Supplementary Figure 17.** **Recombinant glycoprotein Ib blocks rEIII induced**-**platelet pyroptosis.** The rEIII –platelet binding (A) and rEIII (0.6 μM)- induced platelet cell death (B, C) and be blocked by BSA, soluble recombinant CLEC5A (rCLEC5A), glycoprotein Ib (rGP1bα) (10 μg/mL) (B, C). In addition, anti- GP1bα antibody can induce platelet cell death (D, E), in which the dosages used for anti- GP1bα antibody are indicated, 1 fold (1×) = 5 μg/mL, 2 fold (2×) = 10 μg/mL, 4 fold (4×) = 20 μg/mL (D). The cell-type-specific RCD patterns (CTS-RCDPs) of mouse platelet (PLT), after stimulation by anti- GP1bα antibody, DENV and rEIII, respectively, are showed (E). Similar to the RCDs induced by DENV and rEIII, pyroptosis (blue) is the major platelet RCD induced by anti- GP1bα antibody (E). n = 3. ** *P* < 0.01, vs. respective vehicle groups (B, C); ** *P* < 0.01, vs. untreated groups (D).
